# Directly optimizing for synthesizability in generative molecular design using retrosynthesis models†

**DOI:** 10.1039/d5sc01476j

**Published:** 2025-03-21

**Authors:** Jeff Guo, Philippe Schwaller

**Affiliations:** a École Polytechnique Fédérale de Lausanne (EPFL) Switzerland jeff.guo@epfl.ch philippe.schwaller@epfl.ch; b National Centre of Competence in Research (NCCR) Catalysis Switzerland

## Abstract

Synthesizability in generative molecular design remains a pressing challenge. Existing methods to assess synthesizability include heuristics-based metrics or retrosynthesis models which predict a synthetic pathway. By contrast, an explicit approach anchors generation with “synthetically-feasible” chemical transformations, such that all generated molecules already have a predicted synthetic pathway. To date, retrosynthesis models have been mostly used as a *post hoc* filtering tool as their inference cost remains prohibitive to use directly in an optimization loop. In this work, we show that with a sufficiently sample-efficient generative model, it is straightforward to directly optimize for synthesizability using retrosynthesis models in goal-directed generation. Under a heavily-constrained computational budget, our model can generate molecules satisfying multi-parameter drug discovery optimization tasks while being synthesizable, as deemed by retrosynthesis models. We reaffirm previous findings that common synthesizability heuristics (formulated based on known bio-active molecules) can be well correlated with retrosynthesis models' solvability, such that optimizing for the latter may not be an optimal allocation of computational resources. However, going further, we show that moving to other classes of molecules, such as functional materials, current heuristics' correlations diminish, such that there is an advantage to incorporating retrosynthesis models directly in the optimization loop. Finally, we demonstrate that over-reliance on synthesizability heuristics can overlook promising molecules. The codebase is available at https://github.com/schwallergroup/saturn.

## Abstract 2

Existing approaches to consider the synthesizability of generated molecules. This work demonstrates the use of an explicit retrosynthesis model directly as an optimization objective.

## Graphical abstract

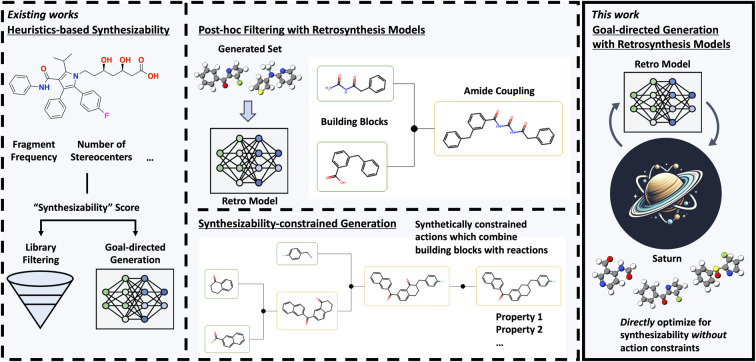

## 1 Introduction

Generative molecular design for drug discovery has achieved experimental validation across numerous targets, with many candidate molecules progressing into clinical trials.^1^ However, the synthesizability of generated designs remains a pressing challenge. Regardless of how “good” generated molecules are, they must be synthesized and experimentally validated to be of use, and work has shown that many generative models propose molecules for which finding a viable synthetic route is, at the very least not straightforward.^2,3^

Existing works tackle synthesizability in generative molecular design either by incorporating synthesizability metrics (*via* heuristics^4^ or retrosynthesis models^5^) in the objective functions or by explicitly enforcing a notion of synthesizability directly in the generative process.^6–8^ The latter can be broadly categorized as synthesizability-constrained generative models and have become increasingly prevalent. A typical metric to quantify synthesizability is whether a retrosynthesis model can solve a route for the generated molecules. While synthesizability-constrained generative models already, by design, output a predicted synthetic pathway, recent works have additionally applied retrosynthesis models for *post hoc* assessment.^9–11^ It is common practice to apply retrosynthesis models during *post hoc* filtering due to their inference cost.^2,3^ On the other hand, sample efficiency is also a pressing challenge, which concerns with how many oracle calls (computational predictions of molecular properties) are required to optimize an objective function. When these oracle calls are computationally expensive, there is a practical limit to an acceptable oracle budget for real-world model deployment. The Practical Molecular Optimization (PMO) benchmark^12^ highlighted the importance of sample efficiency and since then, more recent works have explicitly considered an oracle budget.^13–22^

Saturn,^22^ a language-based molecular generative model built on the Mamba architecture,^23^ has recently demonstrated state-of-the-art sample efficiency when compared to 22 existing models. Building on this advancement, our work shows that with a sufficiently sample-efficient generative model, retrosynthesis models can be treated as oracles and directly incorporated into the molecular generation optimization process – specifically targeting molecules with feasible synthesis routes. Although similar approaches have been explored,^24–26^ our work explores optimization under a significantly more constrained oracle budget (1000 evaluations compared to their 32 000, 64 000, and 256 000 on the hardest task, respectively). We also perform multi-parameter optimization (MPO) involving docking and semi-empirical quantum-mechanical simulations. These computations can be expensive, thus understanding optimization under these settings is practically important. Our contribution is as follows:

(1) Contrary to many existing works, we directly optimize for synthesizability using any (template-based, graph edits-based, seq2seq SMILES) retrosynthesis model. Our approach can outperform specialized synthesizability-constrained generative models on MPO tasks and generate more desirable molecules under highly constrained computational budgets.

(2) We intentionally pre-train a model unsuitable for generating synthesizable molecules and present an optimization recipe leveraging synthesizability heuristics that can fine-tune this model to generate synthesizable molecules in under a minute (building on observations made in previous works^2,27,28^).

(3) We show that when moving from “drug-like” molecules to functional materials, the ability to leverage the correlation between synthesizability heuristics and retrosynthesis models' solvability is diminished. In these cases, directly optimizing for retrosynthesis models can offer clear benefits.

(4) Finally, it is well known that synthesizability heuristics are imperfect in predicting synthesizability and that “poor” heuristic scores can still entail synthesizable molecules.^4^ We supplement existing works and explicitly show that in the generative paradigm, incorporating retrosynthesis models directly in the optimization loop can highlight desirable chemical spaces that would have been overlooked had only heuristics scores be considered.

## 2 Related works

### 2.1 Assessing synthesizability

#### 2.1.1 Synthesizability metrics

Quantifying and defining synthesizability is non-trivial and early metrics assess molecular complexity rather than synthesizability explicitly. Exemplary works include the Synthetic Accessibility (SA) score^4,29^ and SYnthetic Bayesian Accessibility (SYBA)^30^ which are based on the frequency of chemical groups in databases. The Synthetic Complexity (SC) score^31^ is trained on Reaxys data to measure molecular complexity and implicitly considers the number of synthetic steps required to make a target molecule. There is a correlation between these scores and whether retrosynthesis tools can solve a route.^2,27,28,32^ The recent Focused Synthesizability (FS) score^33^ incorporated domain-expert preferences^34^ to assess synthesizability.

#### 2.1.2 Retrosynthesis models

Given a target molecule, retrosynthesis models propose viable synthetic routes by combining commercial building blocks (starting reagents) with reaction templates (coded patterns that map chemical reaction compatibility) or template-free approaches (learned patterns from data). Exemplary examples include the first works^35–37^ targeting retrosynthesis and applying Monte Carlo tree search (MCTS).^5^ Retrosynthesis platforms include SYNTHIA,^38,39^ AiZynthFinder (AiZynth),^40,41^ ASKCOS,^37,42,43^ Eli Lilly's LillyMol,^44^ Molecule.one's M1 platform,^45^ and IBM RXN.^46–48^ We further highlight surrogate models including Retrosynthesis Accessibility (RA) score^49^ and RetroGNN^50^ trained on the output of retrosynthesis models for faster inference. Note that these models output a score rather than synthetic routes. Finally, recent work proposes bidirectional synthesis planning, allowing starting material constraints.^51^

### 2.2 Generating synthesizable molecules

#### 2.2.1 Enumeration-based methods

Expansion methods include SYNOPSIS,^52^ Design of Genuine Structures (DOGS),^53^ design of innovative NCEs generated by optimization strategies (DINGOS),^54^ and RENATE.^55,56^ These methods enumerate candidate molecules following a set of pre-defined reaction rules and have demonstrated experimental validation.^56^

#### 2.2.2 Machine learning approaches

More recently, machine learning methods encompassing molecular generative models have been formulated with a notion of explicit synthesizability which we categorize broadly into synthesizability-constrained models and goal-directed generation with synthesizability metrics.

#### 2.2.3 Synthesizability-constrained molecular generation

Synthesizability-constrained generative models explicitly constrain generation by enforcing transformations from predicted reactivity of building blocks or from a set of permitted reaction templates. These methods include MOLECULE CHEF,^6^ Synthesis Directed Acyclic Graph (DAG),^7^ ChemBO,^57^ SynNet,^8^ SyntheMol,^58^ SynFormer,^59^ and SynthFormer.^60^ Models that also use reinforcement learning (RL) include Policy Gradient for Forward Synthesis (PGFS),^61^ Reaction-driven Objective Reinforcement (REACTOR),^62^ LibINVENT,^63^ and SynFormer.^59^ Recent works have equipped GFlowNets^64^ with reaction templates, including SynFlowNet,^10^ RGFN,^9^ and RxnFlow.^11^ Recent work proposes a model capable of “projecting” unsynthesizable molecules into similar, but synthesizable analogs.^65^

#### 2.2.4 Goal-directed generation with synthesizability metrics

**Fig. 1:**
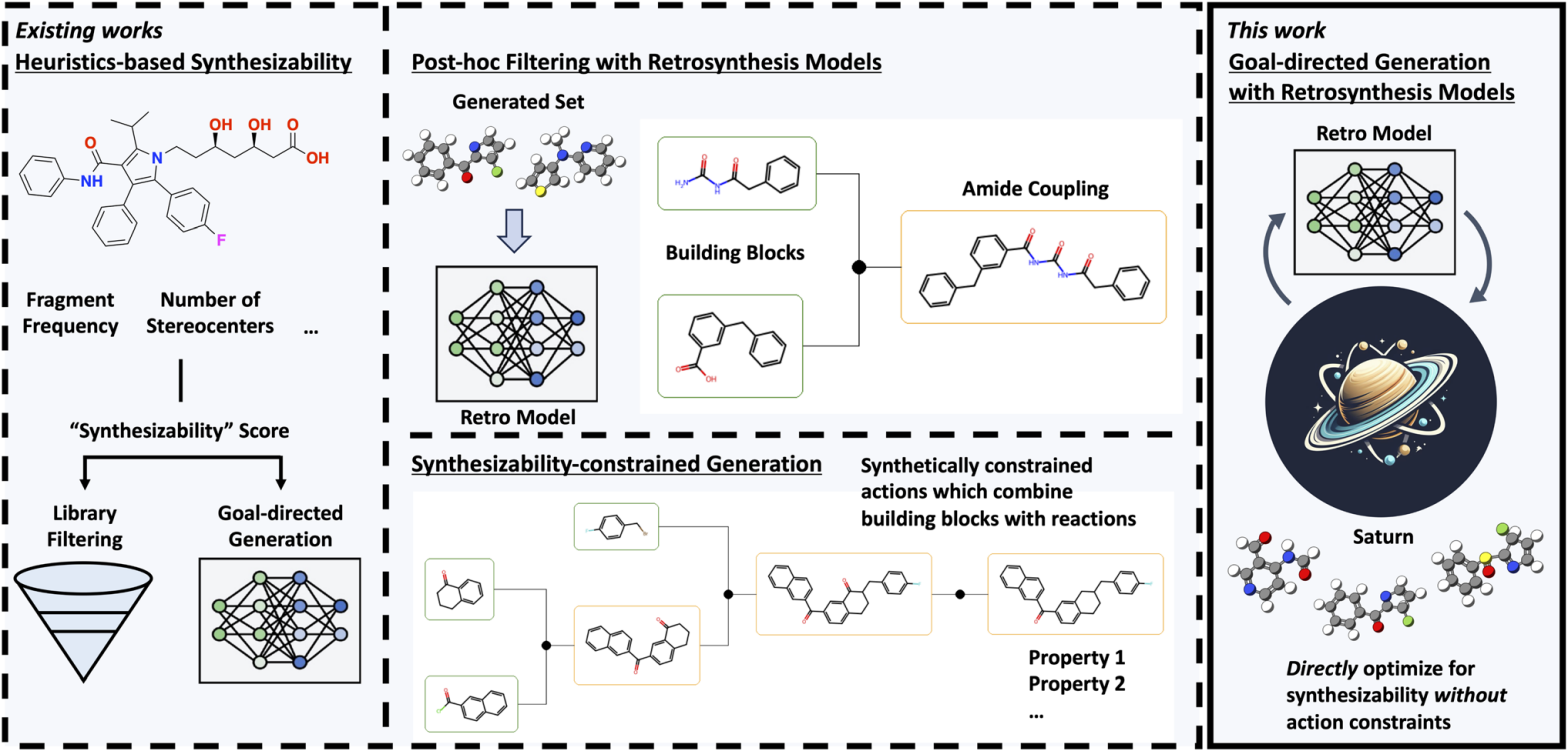
Overview of algorithmic methods to handle synthesizability in generative molecular design.

An alternative to synthesizability-constrained molecular generation is to task molecular generative models to also optimize for synthesizability metrics^33,66^ in the objective function, with common ones being SA score.^2,3^ Although the SA score assesses molecular complexity, it is correlated with whether AiZynth can solve a route.^2,27,28,32^ Generally, more confidence is placed on the output of retrosynthesis models in assessing synthesizability, which is reflected in recent studies that quantify model performance based on whether generated molecules have a solved route.^9–11,65^ Here, we incorporate retrosynthesis tools as an oracle in MPO objective functions to directly optimize for synthesizability (Fig. 1).

## 3 Methods

In this section, we describe in detail the generative model, retrosynthesis models used, and the case studies (drug discovery and functional materials). We defer experiment-specific details including objective functions and metrics to the corresponding Results sections.

### 3.1 Unconstrained generative model

We build on Saturn^22^ which is an autoregressive language-based molecular generative model using RL. In that work, we demonstrated state-of-the-art sample efficiency in dense reward environments, where every molecule gave at least some reward. However, it is unclear the performance of Saturn when high reward molecules are more sparse and this can be a challenge when including retrosynthesis models as an oracle. In particular, retrosynthesis models either find a synthetic route or not, and rewarding partially decomposed target molecules is not necessarily chemically sound, *i.e.*, just because a molecule can be partially broken down, does not mean that molecule is “more” synthesizable given fixed building blocks and reaction rules. In this study, we show that this increased sparsity, while making optimization more challenging, can still be learned. Finally, Saturn is separately pre-trained on the common ChEMBL 33 (ref. 67) or ZINC 250k^68^ datasets to show that regardless of the pre-training dataset, suitable molecules can be generated in reasonable time (we report wall times).

### 3.2 Retrosynthesis models

Saturn's optimization loop is agnostic to the specific retrosynthesis model used. In an effort to demonstrate this behavior, we use four retrosynthesis models in this work, which span distinct formulations. AiZynthFinder (from here on, written as AiZynth)^40,41^ uses reaction templates and MCTS search. The recent Syntheseus^69^ library benchmarked and provides access to numerous retrosynthesis models, showing trade-offs in accuracy and speed. Subsequently, we also use the RetroKNN (template-based),^70^ Graph2Edits (graph edits-based),^71^ and RootAligned (seq2seq SMILES-based)^72^ retrosynthesis models. We chose these models because they are amongst the best accuracy and speed (as benchmarked in Syntheseus) and cover diverse classes of model formulations. In contrast to AiZynth which uses MCTS, we use the Retro* search algorithm^73^ for these models to emphasize that our approach is retrosynthesis model-agnostic. We used the default hyperparameters for all models. The maximum permitted search time for AiZynth and Syntheseus models were 120 and 180 seconds, respectively, and was terminated if one valid route is found. Lastly, retrosynthesis models propose synthetic routes based on a pre-defined set of “building blocks” (it is common to consider commercially available reagents). We use several building block stocks (size in parentheses) in the retrosynthesis models including AiZynth's default stock which is based on ZINC^40,41,68^ (17 422 831), the ‘Fragment’ and ‘Reactive’ sub-sets of ZINC (17 721 980), Enamine US stock (249 946), Enamine EU stock (160 244), and the Sigma Aldrich sub-set from the original ASKCOS^42^ work (20 290). We use different building block stocks with varying sizes to provide commentary on the effects. In each experiments' section, we specify which stock was used.

### 3.3 Drug discovery case study

We use the case study proposed in the recent synthesizability-constrained Reaction GFlowNet (RGFN)^9^ work and re-implement their case study faithfully as the code was not released (see the ESI† for details). We use this case study in experiments to compare to their work and to showcase that our approach is retrosynthesis model-agnostic. The case study is to generate molecules with optimized QuickVina2-GPU-2.1 (ref. 74–76) docking scores against ATP-dependent Clp protease proteolytic subunit (ClpP)^77^ while being synthesizable, as deemed by the AiZynth^40,41^ retrosynthesis model, *i.e.*, whether a synthetic route can be found.

### 3.4 Functional materials case study

We follow the case study proposed by Yuan *et al.*^78^ to design potential organic semiconductors by targetting specific ranges of electronic properties. Using semi-empirical quantum-mechanical calculations through xTB,^79^ the task is to generate molecules with 1.8 eV ≤ HOMO–LUMO gap ≤ 2.2 eV and dipole moment < 2 Debye. We additionally add the constraint that generated molecules must be solvable by a retrosynthesis model.

## 4 Results and discussion

We devise six experiments to answer specific questions. The specific questions are discussed in the corresponding Results sections:

(1) (Drug discovery): optimizing only the docking score following the RGFN work.^9^

(2) (Drug discovery): jointly optimizing docking score and AiZynth.

(3) (Drug discovery): jointly optimizing docking score and AiZynth starting from an intentionally unsuitable pre-trained model.

(4) (Drug discovery): jointly optimizing docking score and synthesizability using any retrosynthesis model.

(5) (Drug discovery and functional materials): demonstrating that going “out-of-distribution” of synthesizability heuristics can lead to misleading proxies of synthesizability

(6) (Drug discovery): demonstrating that over-reliance on synthesizability heuristics can overlook promising chemical spaces.

In experiments 1 and 2, we compare to previous models: GraphGA,^80^ SyntheMol,^58^ FGFN,^81^ and RGFN.^9^ We highlight one caveat in the ensuing comparisons: Saturn is pre-trained with the common ChEMBL 33 (ref. 67) or ZINC 250k^68^ datasets which contain bio-active molecules and inherently bias the learned distribution to already known synthesizable entities.^2^ On the other hand, synthesizability-constrained models, such as RGFN defines a state space based on reaction templates and building blocks. Therefore, the starting distribution is not the same. However, we believe this is still a meaningful comparison because we pre-train on common datasets, used in all generative molecular design benchmark literature.^12,82,83^ Finally, all experiments with the exception of experiment 1, were run across 10 seeds (0–9 inclusive) and the mean and standard deviation are always reported.

### 4.1 Experiment 1: optimizing only docking score leads to unreasonable molecules

**Fig. 2:**
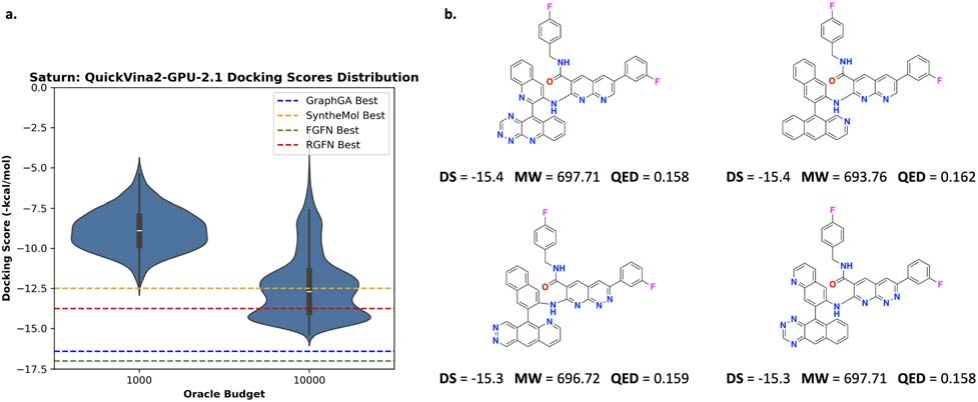
Experiment 1: optimizing only docking scores. (a) Distribution of docking scores at varying oracle budgets. The estimated best docking score across comparison methods (estimated from Fig. 3 in the RGFN^9^ work) are annotated as dotted lines. (b) Example lipophilic molecules generated by Saturn with the best docking scores. Based on the RGFN work, all models except Saturn were run with a 400 000 oracle budget or 72 hours, whichever was reached first. For Saturn, we used a 1000 or 10 000 oracle budget.

In this section, we follow RGFN's^9^ case study exactly and show that only optimizing for docking scores can negatively compromise physico-chemical properties. Correspondingly, we use the following reward function:

1 *R*_RGFN_(*x*) = Docking score(*x*)

where *x* is a generated molecule. It is generally not advised to optimize this in isolation because docking oracles can be highly exploitable, such that lipophilic (lots of carbon atoms and high log *P*) molecules (promiscuous binders with solubility issues^84^) receive good docking scores. We show that with 10 000 oracle calls, Saturn (pre-trained on ChEMBL 33) generates molecules with approximately the same best docking scores compared to GraphGA,^80^ SyntheMol,^58^ FGFN,^81^ and RGFN^9^ which were run with 400 000 oracle calls (40× higher budget). We perform one replicate here as we only want to convey that the objective function is highly exploitable. Fig. 2a shows the distribution of docking scores at varying oracle budgets. We illustrate how the docking oracle can be exploited in Fig. 2b which shows the best molecules generated by Saturn. Although possessing good docking scores, they are lipophilic with high molecular weight and low QED. Consequently, these are not meaningful molecules. Table 1 in the RGFN^9^ work shows that the best generated molecules across various models also have low QED: GraphGA (∼0.32), FGFN (∼0.22), and RGFN (∼0.23), suggesting that they are also exploiting the docking oracle. We note that SyntheMol has slightly higher QED (∼0.45).

**Table 1:** Synthesizability metrics for top-k Modes (molecules with docking score < −10). Results are taken from the RGFN^9^ work (it was not stated how many replicates the models were run for). Mol. weight, QED, and SA score are reported for the top-500 Modes while AiZynth is for the top-100 Modes. All Saturn experiments were run across 10 seeds (0–9 inclusive). The mean and standard deviation are reported. Both Yield and Modes are reported. The number after the configuration denotes the number of successful replicates out of 10 (Modes ≥ 1). For Saturn, none of the configurations found 100 Modes in 1000 molecules generated so the metrics are reported for however many Modes were found. NR denotes “not reported”

Method	Modes (yield)	Mol. weight (↓)	QED (↑)	SA score (↓)	AiZynth (↑)	Molecules generated (wall time)
**Previous work**		**Top-500**	**Top-500**	**Top-500**	**Top-100**	
GraphGA	NR	521.0 ± 31.8	0.32 ± 0.07	4.14 ± 0.51	0.00	400 000 (NR)
SyntheMol	NR	458.2 ± 60.7	0.45 ± 0.16	2.86 ± 0.56	0.56	100 000 (72 h)
FGFN	NR	548.6 ± 42.9	0.22 ± 0.03	2.94 ± 0.54	0.25	400 000 (NR)
RGFN	NR	526.2 ± 37.6	0.23 ± 0.04	2.83 ± 0.22	0.65	400 000 (72 h)

***R***_**All MPO**_ **(ours)**	**4 Objectives (docking, QED, SA, AiZynth)**					
Saturn-ChEMBL (10)	7 ± 3 (16 ± 13)	359.3 ± 19.2	0.73 ± 0.08	2.08 ± 0.12	0.89 ± 0.15	1000 (2 h 35 m ± 12 m)
Saturn-ZINC (10)	5 ± 4 (8 ± 5)	369.9 ± 44.0	0.76 ± 0.09	2.28 ± 0.41	0.94 ± 0.10	1000 (2 h 25 m ± 12 m)

***R***_**Double MPO**_ **(ours)**	**2 Objectives (docking, AiZynth)**					
Saturn-ChEMBL (10)	59 ± 12 (170 ± 45)	420.9 ± 24.9	0.40 ± 0.04	2.25 ± 0.11	0.90 ± 0.05	1000 (2 h 35 m ± 21 m)
Saturn-ZINC (10)	26 ± 11 (81 ± 47)	415.7 ± 24.1	0.52 ± 0.08	2.36 ± 0.22	0.94 ± 0.06	1000 (2 h 31 m ± 10 m)

### 4.2 Experiment 2: directly optimizing for synthesizability using AiZynth

In the previous section, we have shown that generative models can exploit docking oracles. Yet, docking scores can be valuable as they can be correlated with better binding affinity^85^ and should be optimized in combination with oracles that modulate physico-chemical properties. Correspondingly, we define two reward functions in this section:2
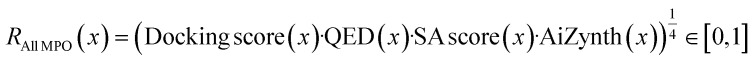
3
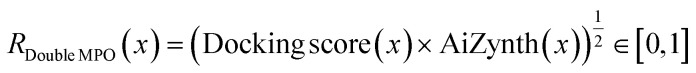


See the ESI† for details on reward shaping to normalize eqn (2) and (3) ∈ [0, 1] and the exponential terms from the product aggregator that outputs the final reward. The rationale for *R*_All MPO_ (eqn (2)) is because RGFN evaluates generated molecules also by their quantitative estimate of “drug-likeness” (QED) score^86^ and SA score.^4^ Since these are the downstream metrics, we include them in the objective function. The rationale for *R*_Double MPO_ is to illustrate a contrast in optimization difficulty as *R*_All MPO_ is inherently more challenging. Moreover, the use of *R*_Double MPO_ is to explicitly convey a sub-message of this section: if certain properties are desirable in the generated molecules, then these properties should be explicitly optimized for. Since *R*_Double MPO_ does not optimize for QED, we do not expect the generated molecules to possess high QED. We verify this in the results. Finally, since synthesizability heuristics are computationally inexpensive, we perform an additional set of experiments coupling GraphGA and Saturn which are non-synthesizablity-constrained models with SA score (and QED) and run *post hoc* filtering with AiZynth for comparison. The corresponding reward function is:4
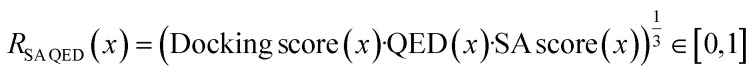


#### 4.2.1 Metrics

Following the RGFN^9^ work, Modes is defined as the set of molecules with docking score < −10 that also possess Tanimoto similarity < 0.5 to each other. We note that this threshold is somewhat arbitrary, so for Saturn results, we additionally report Yield which denotes the total number of unique molecules generated with a docking score < −10.

#### 4.2.2 Experimental set-up

For the *R*_All MPO_ and *R*_Double MPO_ experiments, we use an oracle budget of 1000 calls (how many molecules are assessed by the objective function). While RGFN used an oracle budget of 400 000 calls, an exact apples-to-apples comparison on oracle budget alone cannot be made. This is because our approach incorporates a retrosynthesis model directly in the optimization loop, which is considerably more computationally expensive than just docking used in RGFN. Therefore, we report the wall times as RGFN enforced a limit of 72 hours and also refer to “oracle calls” in just this section, as “molecules generated”. We compare our approach with GraphGA,^80^ SyntheMol,^58^ Fragment-based GFlowNet (FGFN),^81^ and RGFN.^9^ For the *R*_SA QED_ experiments with GraphGA and Saturn,^22^ we use 3000 and 1000 oracle budgets, respectively. We do this so that the wall times of the models are similar. We continue to enforce the highly constrained oracle budget even though the runs themselves are not particularly long because: (1) these models are sample-efficient^12,22,87^ and (2) should the oracle be more expensive, increasing oracle budgets become less feasible.^12^

#### 4.2.3 Quantitative results

Table 1 shows the Saturn results and also results taken from RGFN's^9^ work. The central message of this section is that molecules satisfying the objective functions can be found within 1000 generated molecules. An important note is that all RGFN results (top half of Table 1) report results for the top-500 (for Mol. weight, QED, SA score) and top-100 (for AiZynth) Modes. Saturn does not find 100 Modes in all configurations with 1000 generated molecules so the metrics are reported for however many Modes were found. Regardless of the pre-training dataset (ChEMBL 33 or ZINC 250k), Saturn can generate molecules satisfying *R*_All MPO_ and *R*_Double MPO_. We make the following specific observations: by including AiZynth in the objective function, AiZynth solvable molecules are generated. Including QED and SA score in the objective function also optimizes these metrics (contrast *R*_All MPO_ with *R*_Double MPO_ results). *R*_Double MPO_ finds notably more Modes than *R*_All MPO_ because the optimization task is easier. Finally, we highlight that although the raw number of Modes generated when using the *R*_All MPO_ objective function is relatively low (in 1000 generated molecules), if AiZynth does accurately predict “true” synthesizability, then these Modes are immediately actionable. Importantly, they satisfy every metric in the objective function (low docking score, high QED, low SA score, and is AiZynth solvable). See the ESI† for additional experiments showing that optimizing also for QED is a considerably more difficult task.

Next, we comment on the *R*_SA QED_ results that remove AiZynth from the optimization loop and rely on SA score to guide the model towards AiZynth-solvable molecules. Table 2 contrasts the performance between GraphGA and Saturn. We first note that both methods used ZINC 250k to initialize the models. In the case of GraphGA, the initial population was sampled from ZINC 250k, which follows the original publication.^80^ In the case of Saturn, we used the ZINC 250k pre-trained model. Next, we ran GraphGA under two oracle budgets: 1000 and 3000 to compare with Saturn with a 1000 oracle budget. The reason is because GraphGA runs faster than Saturn which requires back-propagation and QuickVina2-GPU is a computationally light docking oracle. Therefore, we increase the oracle budget of GraphGA accordingly so that the wall times of the methods are the same, for maximum fairness. However, this makes two assumptions: (1) there are no additional costs for running more oracle calls, *e.g.*, monetary costs if using API-services or licensed software. (2) We ignore the computational cost of manually running AiZynth on the generated molecules. If one wants to assess more molecules, then the wall time is non-negligible since AiZynth is by far the most computationally intensive oracle compared to GPU docking, QED, and SA score. Note that we refer to “oracle budget” now because the reward function is fixed here and neither model incorporates the retrosynthesis model directly in the optimization loop. Lastly, the hyperparameters of GraphGA were based on the optimal set found in the PMO benchmark.^80^ Based on the results in Table 2, we make the following observations: firstly, Saturn with 1000 oracle calls outperforms GraphGA with 1000 oracle calls across all metrics. Secondly, GraphGA with 3000 oracle calls generates more Modes than Saturn across both docking score thresholds. However, the property values are less optimized, demonstrating that Saturn performs MPO to a much greater extent. As we show in the ESI,† jointly optimized QED makes achieving lower docking scores more difficult because it constraints the molecular weight. One can verify this by contrasting the average molecular weights of the generated molecules. Furthermore, because GraphGA generated molecules are larger, they also have longer synthetic routes (about 1 step longer) as predicted by AiZynth (Table 2). Lastly, the solve rate of Saturn generated molecules are also higher because SA score is optimized to a greater extent, though the absolute number of solved molecules is higher in GraphGA with 3000 oracle calls. Overall, the results convey two things: (1) SA score is a meaningful heuristic for AiZynth-solvability. (2) Saturn optimizes MPO objectives to a greater extent than GraphGA.

**Table 2:** Synthesizability metrics for all Modes using *R*_SA QED_ which does not include AiZynth in the optimization loop. A baseline is also included – Saturn Dock which only optimizes for docking. The metrics are divided based on docking score thresholds. ZINC 250k was used for both GraphGA (initial population sampling) and Saturn (pre-training). All experiments were run across 10 seeds (0–9 inclusive). The mean and standard deviation are reported. Both Yield and Modes are reported. The number after the configuration denotes the number of successful replicates out of 10 (Modes ≥ 1 that are synthesizable). ‘Rxn steps‘ denotes the number of reaction steps of the generated molecules, as predicted by AiZynth

*R*_SA QED_	3 Objectives (docking, QED, SA)						
Method	Modes (yield)	Mol. weight (↓)	QED (↑)	SA score (↓)	Rxn steps (↓)	AiZynth (↑)	Oracle calls (wall time)
**Docking score < −9**							
GraphGA (10)	30 ± 6 (36 ± 7)	398.4 ± 11.1	0.61 ± 0.04	2.77 ± 0.15	3.50 ± 0.30	0.43 ± 0.11	1000 (3 m 2 s ± 5 s)
GraphGA (10)	221 ± 22 (327 ± 55)	386.4 ± 5.1	0.66 ± 0.02	2.48 ± 0.10	3.43 ± 0.15	0.49 ± 0.04	3000 (9 m 12 s ± 18 s)
Saturn Dock (10)	121 ± 33 (378 ± 85)	406.3 ± 14.3	0.54 ± 0.05	2.57 ± 0.28	3.11 ± 0.40	0.59 ± 0.15	1000 (8 m 6 s ± 33 s)
Saturn (10)	38 ± 13 (83 ± 33)	343.3 ± 6.2	0.80 ± 0.05	2.10 ± 0.10	2.04 ± 0.26	0.85 ± 0.07	1000 (8 m 51 s ± 1 m 36 s)

**Docking score < −10**							
GraphGA (8)	3 ± 2 (3 ± 2)	424.5 ± 27.3	0.50 ± 0.11	2.78 ± 0.42	3.26 ± 0.71	0.50 ± 0.23	1000 (3 m 2 s ± 5 s)
GraphGA (10)	29 ± 8 (35 ± 12)	405.7 ± 12.6	0.61 ± 0.05	2.46 ± 0.20	3.46 ± 0.48	0.53 ± 0.16	3000 (9 m 12 s ± 18 s)
Saturn Dock (10)	45 ± 18 (135 ± 53)	432.8 ± 17.2	0.47 ± 0.05	2.64 ± 0.32	3.39 ± 0.44	0.55 ± 0.17	1000 (8 m 6 s ± 33 s)
Saturn (9)	3 ± 1 (4 ± 2)	379.9 ± 18.8	0.72 ± 0.16	2.26 ± 0.16	2.77 ± 0.84	0.82 ± 0.21	1000 (8 m 51 s ± 1 m 36 s)

As an additional experiment, we also run a Saturn configuration optimizing only for docking. As demonstrated previously (Fig. 2), this is prone to docking score exploitation by generating greasy molecules. However, under the limited 1000 oracle calls budget, Saturn only begins to exploit the oracle. This is demonstrated by the higher MW and lower QED compared to the GraphGA and Saturn configurations which incorporate QED in the objective function. Similar to GraphGA which optimizes QED to a lesser extent and results in higher MW molecules, ‘Saturn Only Docking’ generates larger molecules which makes it easier to achieve a better docking score. The consequence is that generated molecules have, on average, a higher number of reaction steps as predicted by AiZynth (Table 2).

#### 4.2.4 Qualitative results

**Fig. 3:**
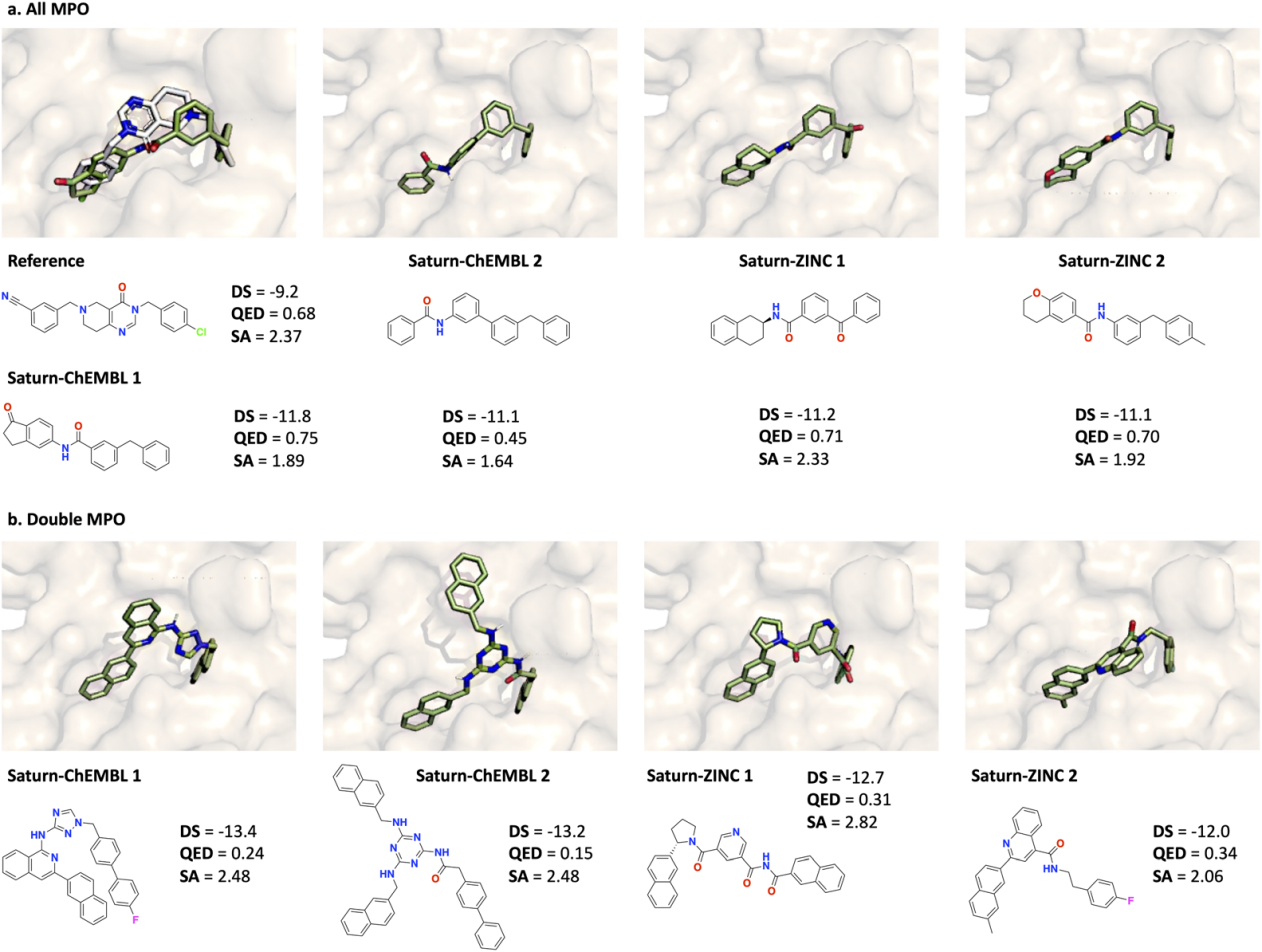
Docked pose of the reference ligand (PDB ID: 7UVU) and generated molecules with the top-2 best docking scores (DS) across all Saturn configurations and across all 10 seeds (0–9 inclusive). The reference pose is in gray and all generated molecules are in green. All molecules are AiZynth solvable. (a) Molecules generated using *R*_All MPO_. (b) Molecules generated using *R*_Double MPO_.

Fig. 3 shows the docking pose for the generated molecules with the top-2 best docking score (no cherry-picking) across all Saturn configurations. In all cases, the pose conforms to the geometry of the binding cavity and the molecule itself is AiZynth solvable (see the ESI† for the solved routes). Generated molecules using *R*_Double MPO_ have better docking scores than *R*_All MPO_, which is expected as the optimization task is easier. In the case of *R*_Double MPO_, the best molecules have docking scores and QED values similar to the best molecules generated by RGFN^9^ in 400 000 oracle calls (Fig. 2). We highlight that the molecules from *R*_Double MPO_ possess extensive carbon rings and are likely exploiting the docking oracle. This is expected because *R*_Double MPO_ does not reward for high QED.

### 4.3 Experiment 3: directly optimizing for synthesizability using AiZynth starting from an unsuitable training distribution

As we have shown in the last part of the previous section, SA score is highly correlated with AiZynth solvability. In this section, we demonstrate a straightforward optimization recipe that allows the generation of AiZynth solvable molecules under minimal computational resources.

Generative models are pre-trained to model the training data distribution. The experiments thus far use Saturn which has been trained with either ChEMBL 33 or ZINC 250k. These datasets contain previously synthesized molecules and pre-trained models can already generate molecules that can be solved by a retrosynthesis model.^2^ In this section, we pre-train Saturn on the fraction of ZINC 250k that is not AiZynth solvable. To do this, we ran AiZynth on the entirety of ZINC 250k and kept the 98 110/248 188 unique SMILES that are not AiZynth solvable. The model pre-trained on this data will be referred to as “Purged ZINC” (see the ESI† for pre-training metrics). The message we convey in this section is that even with an intentionally unsuitable training distribution, both *R*_All MPO_ and *R*_Double MPO_ can still be optimized under a 1000 oracle budget and within 2–3 hours, reinforcing the message that optimizing for synthesizability (as deemed by retrosynthesis models) may not be as difficult as widely believed.

**Fig. 4:**
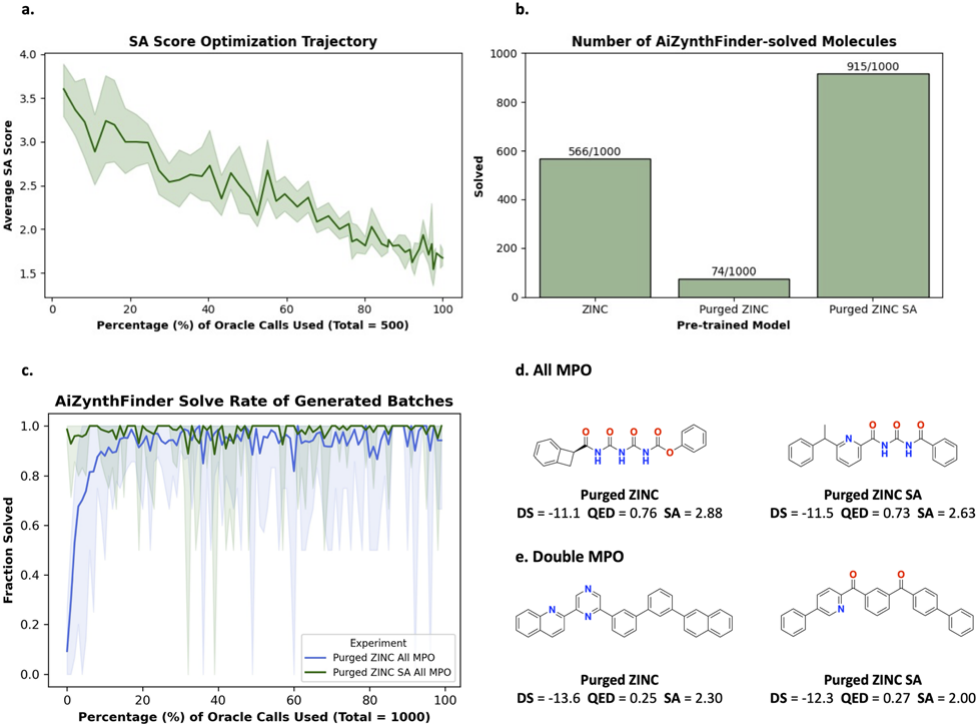
Correlation of SA score and AiZynth solve rate and learning to generate AiZynth solvable molecules. (a) “Purged ZINC” is tasked to minimize SA score. The average SA score of the sampled batches are shown. (b) AiZynth solve rates for 1000 molecules sampled from different models. (c) All MPO task: fraction of generated molecules (without the GA activated) across all batches that are AiZynth-solvable. Values are the mean and the shaded regions are the minimum–maximum across 10 seeds (0–9 inclusive). (d) Example molecules generated from the “Purged ZINC” and “Purged ZINC SA” models with the best docking scores (DS).

To do this, we leverage the correlation of SA score and AiZynth solvability as previously noted.^2,27,28,32^ Specifically, the “Purged ZINC” model should not be expected to generate synthesizable molecules at the start, since it was intentionally pre-trained with non-synthesizable molecules. Tasking such a model to optimize for retrosynthesis solvability immediately would likely result in unsuccessful optimization. Therefore, we apply curriculum learning (CL)^88^ which is a general framework to decompose a complex optimization objective into sequential, simpler objectives. Our end objective is to generate synthesizable molecules with good docking scores. Since the initial model cannot even generate synthesizable molecules, we make the optimization more tractable by first tasking Saturn with only minimizing SA score first. The intended effect is that once the model learns to generate low SA score molecules, it is much more likely that subsequently generated molecules will be AiZynth solvable, precisely because SA score is a noisy proxy for it. Correspondingly, the “Purged ZINC” model is tasked to minimize SA score (500 oracle calls). Fig. 4a shows the optimization trajectory and the resulting model is referred to as “Purged ZINC SA”. The 500 oracle calls are not counted in the 1000 oracle budget, as computing SA score is cheap (this process took 56 seconds). Next, to illustrate distribution learning, we sample 1000 unique molecules from the “Normal ZINC” (trained on the full dataset), “Purged ZINC”, and “Purged ZINC SA” models and run AiZynth. Fig. 4b shows the fraction of molecules that are AiZynth solvable. In under a minute, the “Purged ZINC” model (which generates mostly AiZynth non-solvable molecules) can be fine-tuned to immediately generate molecules that are almost all solvable. Next, we show how the “Purged ZINC” model can learn to generate molecules that are AiZynth solvable during the course of RL (Fig. 4c). We contrast this with the “Purged ZINC SA” model which has almost 100% solve rate throughout the entire run. During the runs, some seeds occasionally generate batches that are not AiZynth solvable (lower bound of the shaded region), but this is not detrimental (see the ESI† for more details). Fig. 4d shows the molecules with the best docking score generated across all seeds. The property profiles are essentially the same as the runs with the “Normal ZINC” model (Fig. 3).

#### 4.3.1 Quantitative results

Table 3 contrasts the results using the “Normal ZINC”, “Purged ZINC”, and “Purged ZINC SA” models. Despite an unsuitable training distribution, “Purged ZINC” can still generate Modes that are AiZynth solvable, although the solve rate can be slightly lower than “Normal ZINC”. “Purged ZINC SA” was first fine-tuned to minimize SA score and already generated mostly AiZynth solvable molecules (Fig. 4b). This process benefits both *R*_All MPO_ (less so) and *R*_Double MPO_ as the Yield and Modes found are higher. Overall, we show that regardless of the starting model, generated molecules with all models possess property profiles better than GraphGA,^80^ SyntheMol,^58^ FGFN,^81^ and RGFN^9^ (contrast Saturn metrics in Fig. 4d compared to the average metrics of the comparing models in Tables 1 and 2).

**Table 3:** Synthesizability metrics for “Normal ZINC” (results from Table 1), “Purged ZINC”, and “Purged ZINC SA”. All experiments were run across 10 seeds (0–9 inclusive). The mean and standard deviation are reported. Both Yield and Modes are reported. The number after the configuration denotes the number of successful replicates out of 10 (Modes ≥ 1 that are synthesizable). The metrics are reported for however many Modes were found

Method	Modes (yield)	Mol. weight (↓)	QED (↑)	SA score (↓)	AiZynth (↑)	Oracle calls (wall time)
***R***_**All MPO**_	**4 Objectives (docking, QED, SA, AiZynth)**					
Normal ZINC (10)	5 ± 4 (8 ± 5)	369.9 ± 44.0	0.76 ± 0.09	2.28 ± 0.41	0.94 ± 0.10	1000 (2 h 25 m ± 12 m)
Purged ZINC (10)	6 ± 5 (9 ± 10)	342.6 ± 14.8	0.75 ± 0.10	2.16 ± 0.25	0.89 ± 0.16	1000 (2 h 49 m ± 20 m)
Purged ZINC SA (8)	13 ± 8 (28 ± 18)	355.2 ± 12.2	0.72 ± 0.05	2.01 ± 0.12	0.97 ± 0.03	1000 (2 h 42 m ± 22 m)

***R***_**Double MPO**_	**2 Objectives (docking, AiZynth)**					
Normal ZINC (10)	26 ± 11 (81 ± 47)	415.7 ± 24.1	0.52 ± 0.08	2.36 ± 0.22	0.94 ± 0.06	1000 (2 h 31 m ± 10 m)
Purged ZINC (10)	31 ± 15 (129 ± 99)	416.4 ± 42.2	0.51 ± 0.13	2.49 ± 0.32	0.89 ± 0.10	1000 (2 h 55 m ± 20 m)
Purged ZINC SA (10)	36 ± 13 (215 ± 133)	426.5 ± 16.2	0.41 ± 0.06	2.20 ± 0.27	0.94 ± 0.08	1000 (3 h 13 m ± 22 m)

### 4.4 Experiment 4: directly optimizing for synthesizability using any retrosynthesis model

Our approach of directly incorporating retrosynthesis models in the optimization loop is retrosynthesis-model agnostic. In the experiments thus far, we used AiZynth.^40,41^ In this section, we run the same *R*_All MPO_ and *R*_Double MPO_ reward functions using RetroKNN (template-based),^70^ Graph2Edits (graph edits-based),^71^ and RootAligned (seq2seq SMILES-based)^72^ as the retrosynthesis models. The building block set is the ZINC ‘Fragment’ and ‘Reactive’ sub-sets (17 721 980) and we use Saturn pre-trained on ChEMBL 33.^67^Table 4 shows the quantitative metrics for *R*_All MPO_ and *R*_Double MPO_. In all cases, the optimization task can be solved within 1000 oracle calls. The wall time discrepancies are due to differences in the retrosynthesis models' inference speed. The wall times may also be longer than AiZynth because we ran multiple experiments simultaneously on a single workstation due to GPU limitations. See the ESI† for more details.

**Table 4:** Synthesizability metrics RetroKNN, Graph2Edits, and RootAligned. All Saturn (pre-trained with ChEMBL 33) experiments were run across 10 seeds (0–9 inclusive). The mean and standard deviation are reported. Both Yield and Modes are reported. The number after the configuration denotes the number of successful replicates out of 10 (Modes ≥ 1 that are synthesizable). For Saturn, none of the configurations found 100 Modes in 1000 oracle calls so the metrics are reported for however many Modes were found

Method	Modes (yield)	Mol. weight (↓)	QED (↑)	SA score (↓)	Solved (↑)	Oracle calls (wall time)
***R***_**All MPO**_	**4 Objectives (docking, QED, SA, Solved)**					
AiZynth (10)	7 ± 3 (16 ± 13)	359.3 ± 19.2	0.73 ± 0.08	2.08 ± 0.12	0.89 ± 0.15	1000 (2 h 35 m ± 12 m)
RetroKNN (10)	3 ± 1 (5 ± 3)	374.0 ± 14.6	0.64 ± 0.12	2.13 ± 0.26	0.67 ± 0.26	1000 (4 h 49 m ± 33 m)
Graph2Edits (7)	5 ± 3 (7 ± 6)	376.2 ± 46.1	0.68 ± 0.05	2.15 ± 0.26	0.88 ± 0.15	1000 (4 h 44 m ± 39 m)
RootAligned (10)	3 ± 1 (5 ± 5)	365.2 ± 22.5	0.70 ± 0.08	2.08 ± 0.13	0.78 ± 0.20	1000 (6 h 8 m ± 52 m)

***R***_**Double MPO**_	**2 Objectives (docking, Solved)**					
AiZynth (10)	59 ± 12 (170 ± 45)	420.9 ± 24.9	0.40 ± 0.04	2.25 ± 0.11	0.90 ± 0.05	1000 (2 h 35 m ± 21 m)
RetroKNN (10)	33 ± 14 (141 ± 88)	444.1 ± 28.5	0.40 ± 0.09	2.26 ± 0.19	0.85 ± 0.06	1000 (3 h 43 m ± 42 m)
Graph2Edits (10)	30 ± 9 (109 ± 65)	446.2 ± 30.2	0.38 ± 0.06	2.29 ± 0.18	0.85 ± 0.11	1000 (3 h 16 m ± 40 m)
RootAligned (10)	31 ± 14 (122 ± 92)	443.6 ± 40.2	0.42 ± 0.08	2.36 ± 0.24	0.81 ± 0.05	1000 (6 h 5 m ± 29 m)

### 4.5 Experiment 5: generating out-of-distribution of synthesizability heuristics

In experiment 2, we showed that optimizing SA score and then *post hoc* filtering with a retrosynthesis model finds more desirable molecules than directly incorporating retrosynthesis models in the optimization loop. The natural question is whether there is any benefit to the latter, as in the end, the desired outcome of a generative experiment is to yield the most synthesizable molecules with the desired property profile. In this section, we answer two questions:

(1) SA score is well correlated with AiZynth solvability with ZINC building blocks. Is this true for other retrosynthesis models, particularly with other (and much smaller) building block stocks?

(2) Thus far, molecules with low SA score tend to be synthesizable. Are there exceptions to this?

#### 4.5.1 Experimental setup

We fix the retrosynthesis model to be RetroKNN^70^ with Retro* search^73^ due to its strong performance in the Syntheseus^69^ benchmark. We consider four building block stocks (size in parentheses):

(1) Enamine EU stock (160 244): Downloaded in November 2024. 1–2 days delivery within the EU.

(2) Enamine US stock (249 946): Downloaded in November 2024. 1–2 days delivery within the US. This stock is commonly employed in synthesizability-constrained generative models.^8,59,65^

(3) Sigma Aldrich (20 290): Downloaded from the original ASKCOS^42^ work.

(4) ZINC ‘Fragment’ and ‘Reactive’ sub-sets (17 721 980): sub-set of AiZynth's^40,41^ building blocks set. This same stock was used in experiment 4.

We perform two case studies and re-iterate the optimization objectives here:

(1) Drug discovery: case study modified from Koziarski *et al.*^9^. Minimize QuickVina2-GPU-2.1 (ref. 74–76) docking scores against ClpP,^77^ maximize QED, minimize SA and/or directly optimize for the retrosynthesis model.

(2) Functional materials: semiconductor: case study based on Yuan *et al.*^78^. Generate molecules with 1.8 eV ≤ HOMO–LUMO gap ≤ 2.2 eV and dipole moment < 2 Debye. Properties computed using xTB (semi-empirical QM).^79^ This will be referred to as the semiconductor case study from here on.

Finally, the goal in this section is to contrast the performance when optimizing for SA score (then *post hoc* filter) *versus* optimizing for retrosynthesis solvability directly. However, computing retrosynthesis solvability takes much longer than computing SA score. Therefore, to ensure a fair comparison, we allocate oracle budgets such that the overall wall time is similar (see ESI†). Correspondingly, for both drug discovery and semiconductor case studies, we run both the retrosynthesis and SA score experiments with a 1000 oracle calls budget and additionally run SA score with either 3000 (drug discovery) or 2000 (semiconductor). The reason why a similar wall time is reached with 2000 SA scores budget in the semiconductor case study compared to 3000 in the drug discovery case study is because xTB is much more expensive to compute than QuickVina2-GPU docking. Therefore, whereas the retrosynthesis model is the main bottleneck in the drug discovery oracle, xTB is non-negligible in the semiconductor oracle. Similar to all previous experiments, we ran every configuration across 10 seeds (0–9 inclusive) and report the mean and standard deviation.

#### 4.5.2 General results

**Fig. 5:**
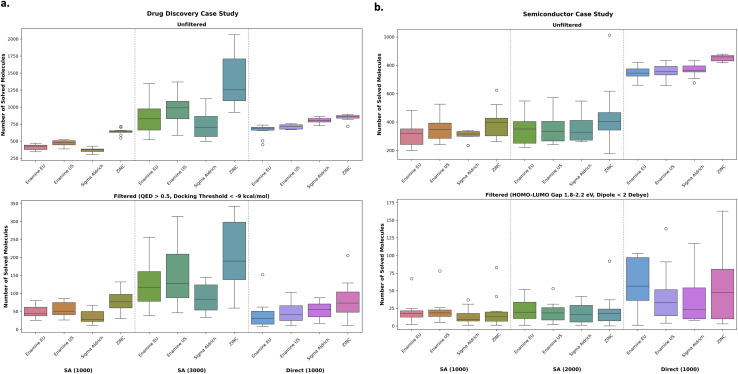
Drug discovery (a) and semiconductor (b) case studies. Comparing SA score optimization and then *post hoc* filtering under various building block stocks or directly optimizing for retrosynthesis solvability. The number in parentheses in the *x*-axes labels represents the number of oracle calls. SA score was run under two different oracle budgets to have a similar wall time with directly optimizing for retrosynthesis solvability (right-hand column in the figures). In the drug discovery (a) case study, it is a better allocation of resources to optimize for SA score. By contrast, in the semiconductor case study, there is a clear benefit in directly optimizing retrosynthesis solvability.

Fig. 5 shows the number of synthesizable molecules across various building block stocks and whether SA score or the retrosynthesis model was incorporated in the optimization loop. Firstly, using the ZINC stock generally results in more synthesizable molecules which is not unexpected, as there are many more building blocks. The other building stocks are comparable. Next, in agreement with the results thus far, it is a better allocation of computational resources to optimize for SA score instead of retrosynthesis model solvability in the drug discovery case study. In both the unfiltered (all results) and filtered (optimal molecules), optimizing for SA score under the same wall time (3000 oracle calls) yields many more synthesizable molecules. By contrast, in the semiconductor case study, directly including the retrosynthesis model in the optimization loop generates more synthesizable molecules. This is especially apparent in the unfiltered case. In the filtered set, this difference diminishes due to the high variance. Running a *t*-test shows that only in the case of Enamine EU are the distributions different (statistically significant at the 95% confidence level). We hypothesize the diminished difference is because the objective is more challenging as the model also needs to ensure the electronic properties are within those intervals. If the oracle budget were increased, the difference may become more stark. This is supported by the unfiltered results which show a clear differentiation. We further support this statement by analyzing the SA score distributions of the semiconductor sets, which show that when optimizing explicitly for SA score, they tend to be lower (as expected), yet, it does not necessarily translate to being more synthesizable (see the ESI†). We find that in the semiconductor case study, SA score and synthesizability (across all stocks) is almost completely uncorrelated (see the ESI†). This suggests that the SA score heuristic is less reliable when moving beyond “drug-like” molecules, since SA score was formulated based on PubChem^89^ molecules.

#### 4.5.3 Semiconductor: effect of building blocks stock and plausibility of generated molecules

**Fig. 6:**
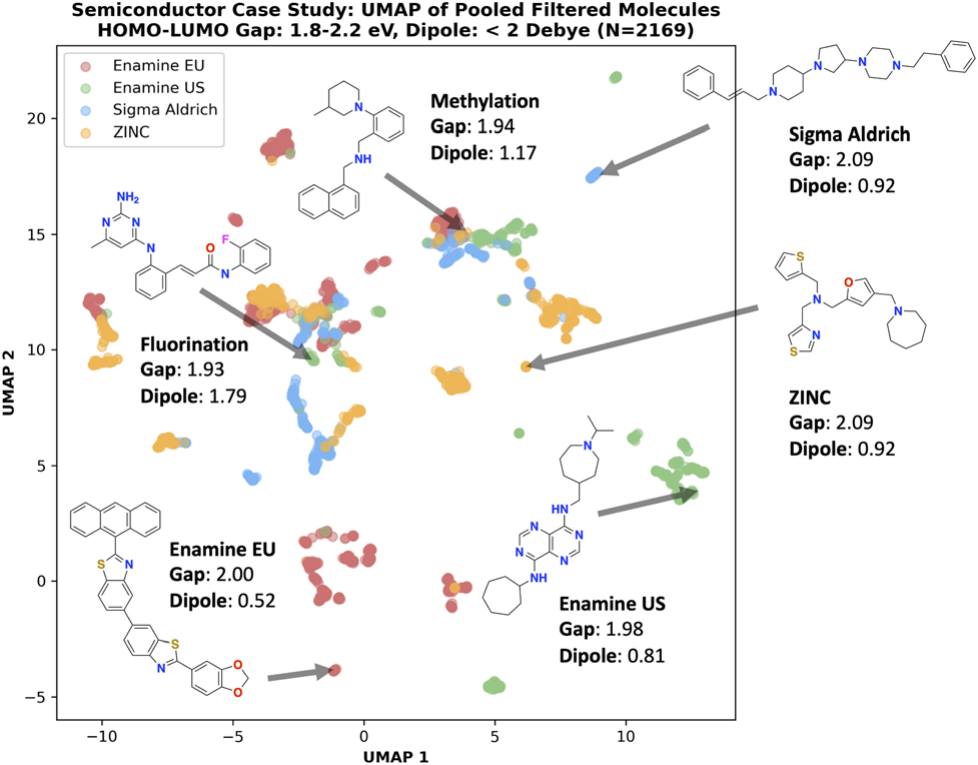
Semiconductor case study: UMAP of pooled filtered (1.8 eV ≤ HOMO–LUMO gap ≤ 2.2 eV and dipole moment < 2 Debye) molecules. Different building block stocks define the synthesizable space and thus generated molecules can occupy distinct chemical space.

Next, we focus on the semiconductor case study and investigate the implications of using different building block stocks. All filtered (1.8 eV ≤ HOMO–LUMO gap ≤ 2.2 eV and dipole moment < 2 Debye) molecules were pooled across all 10 seeds, resulting in 2169 total molecules. Fig. 6 shows the UMAP^90^ of these molecules, colored by building block stock. Building block stocks can occupy different chemical space, showing that changing this parameters leads to different synthesizable molecules. This is not completely unexpected as the building blocks define the synthesizable space. We note that it is possible that if more seeds were performed, this separation becomes less pronounced, but we believe 10 seeds is a reasonable number. Fig. 6 shows four molecules from each stock with an embedding distance far from each other to illustrate a notion of distinctive chemical space. Next, we comment on the plausibility of the generated molecules by highlighting two generated molecules with fluorine and methyl groups. In Yuan *et al.*,^78^ the authors state that fluorination and methylation are common substitutions for these classes of molecules. Moreover, the generated molecules bear similarity to the validated (with DFT) molecules in Yuan *et al.*,^78^ which feature extensive carbon conjugation with nitrogen and sulfur atoms.

### 4.6 Experiment 6: over-reliance on heuristic scores can overlook promising molecules

In the previous section, the overarching message of the drug discovery case study is that optimizing SA score is a better allocation of computational resource than directly including retrosynthesis models in the optimization loop. In this section, we comment deeper on this and start by discussing the implications of the docking oracle. For most drug discovery tasks performed in literature, Autodock Vina docking or its successors are used.^74–76,91^ These docking oracles are often permissive, such that many molecules can achieve a good docking score, with decreased ability to distinguish across protein targets.^85,92^ In commercial drug discovery, it is common practice to employ constrained docking, where for example, specific interactions are to be retained,^85^ which makes designing optimal molecules much more difficult. However, most open-source docking algorithms, like Autodock Vina^74^ and gnina,^91^ do not support this. As a consequence, synthesizability heuristics like SA score can work extremely well as a noisy proxy for retrosynthesis solvability because “optimal” molecules need not deviate from the chemical space of PubChem, and SA score is formulated based on this database. In this section, we aim to explicitly illustrate that there are interesting synthesizable molecules that possess poor SA scores in the drug discovery case study. Our intended message in this section is that over-reliance on optimal SA scores may overlook interesting chemical space, on the basis of molecules with “sub-optimal” SA scores being filtered out.

#### 4.6.1 Experimental setup

We define an “artificial” experiment by using the same drug discovery case study, but instead of minimizing SA score, we maximize it, while incorporating the RetroKNN^70^ with Retro*^73^ retrosynthesis model in the optimization loop. This is “artificial” in the sense that one would likely never want to make synthesizability “harder”. We use the Sigma Aldrich (20 290 building blocks) stock to show that interesting molecules can be overlooked even with this relatively small building block set which defines a smaller synthesizable space. Finally, we use an oracle budget of 10 000 and run the experiment across 5 seeds (0–4 inclusive) and set Saturn to perform more explorative sampling,^22^ to show more diverse scaffolds. We also increased the budget because we want to maximize SA score to above 4, which is generally considered “undesirable” and above the threshold for common *post hoc* filters. We include the oracle budget here for completeness, but it is not important in the discussion as we are not comparing to any models.

#### 4.6.2 “Poor” SA score molecules can be promising

**Fig. 7:**
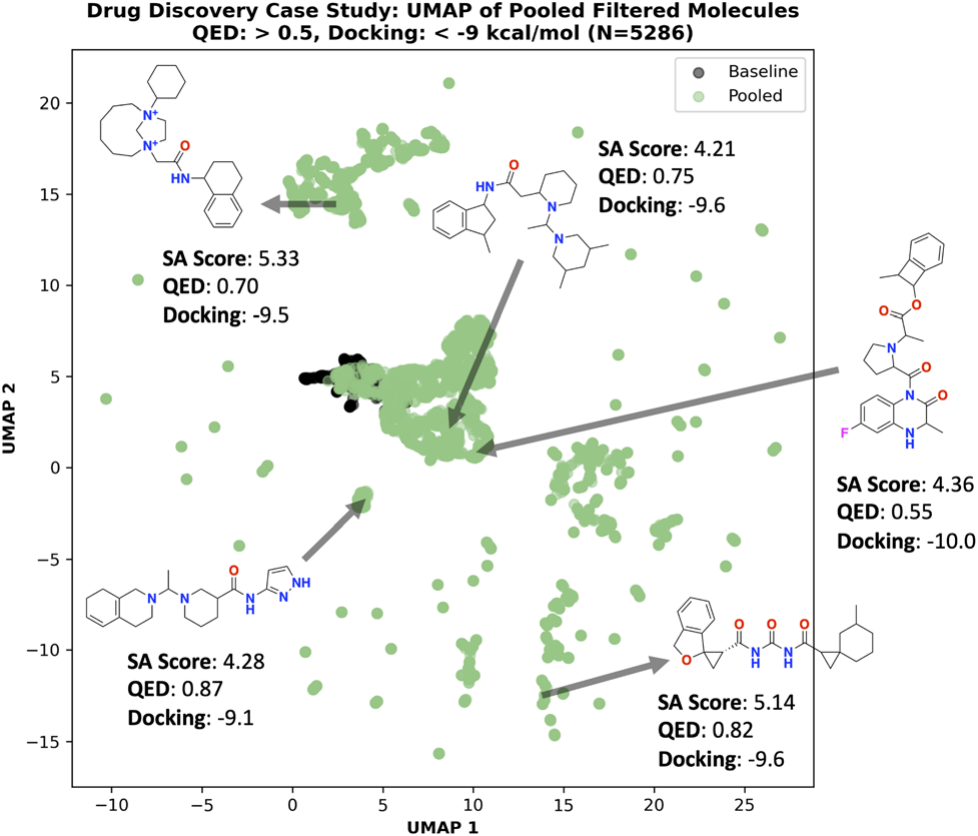
Drug discovery case study: UMAP of pooled filtered (QED > 0.5 and docking score < −9 kcal mol^−1^) molecules. Generated molecules with “poor” SA scores can still be promising. “Baseline” denotes the pooled molecules (10 seeds, 1000 oracle calls budget) generated by minimizing SA score while “Pooled” are the pooled molecules generated by maximizing SA score.

All filtered (QED > 0.5 and docking score < −9 kcal mol^−1^) molecules were pooled across all 5 seeds, resulting in 5286 total molecules. Fig. 7 shows the UMAP^90^ of these molecules compared to the baseline which is the 1000 oracle calls run from the previous section (10 seeds). The molecules generated with “poor” SA scores occupy different chemical space than low SA score molecules, as expected. Example molecules are annotated in Fig. 7 to convey two points: (1) “Poor” SA score molecules can possess rare or undesirable substructures, such as charged atoms and overly small or large rings. (2) “Poor” SA score molecules can also be reasonable molecules that achieve high QED and good docking scores. A specific example is the annotated molecule with pyrazole (aromatic five-membered ring with two nitrogens) as there are many known drugs and bioactive molecules containing this moiety.^93^

Overall, we re-iterate our intended message in this section that molecules with “poor” SA scores can be synthesizable and over-reliance on optimal SA scores can overlook these promising molecules. Naturally, SA score does not provide comprehensive coverage of “drug-likeness” and retrosynthesis solvability can be useful in identifying promising molecules outside the PubChem chemical space.

## 5 Conclusions

In this work, we adapt Saturn^22^ which is a sample-efficient autoregressive molecular generative model using the Mamba^23^ architecture to directly optimize for synthesizability using retrosynthesis models and across drug discovery and functional material case studies. Our approach contrasts existing works in the field that tackle synthesizability in one of three ways: goal-directed generation with synthesizability heuristic scores such as SA score,^4^*post hoc* filtering generated molecules with a retrosynthesis model,^94^ or by enforcing synthesizability design principles in the generative process itself (synthesizability-constrained generation).^8–10^ We show that with a sufficiently sample-efficient model, treating retrosynthesis models as an oracle is feasible, and generated molecules can satisfy multi-parameter optimization objectives while being synthesizable (as deemed by a retrosynthesis model). We compare to recent synthesizability-constrained generative models and show that Saturn can generate synthesizable molecules with optimal property profiles under a heavily constrained oracle budget. By decoupling synthesizability from the generative model itself, we can mix-and-match any retrosynthesis model and we show optimization using a total of four models across different building block stocks of various sizes. Next, we conduct an artificial experiment to intentionally purge a training dataset of all molecules that are solvable by the AiZynthFinder retrosynthesis model and pre-train a new model with this dataset. Generated molecules from this model are mostly not AiZynth solvable, as expected (Fig. 4b). Despite this, we show that within a similar constrained computational budget, this model can still generate molecules with property profiles better than all comparing models and are synthesizable. After this initial set of results, we investigate the interplay between optimizing for synthesizability heuristic scores like SA score compared to retrosynthesis models directly. In the drug discovery case study, we show that optimizing for SA score followed by *post hoc* retrosynthesis model filtering is a better allocation of computational resources. However, in the functional materials case study, we show the opposite effect, as optimizing for retrosynthesis models results in considerably more synthesizable molecules. We hypothesize that materials move outside the distribution of SA score, which is formulated based on the PubChem database. Finally, we illustrate that over-reliance on SA scores can overlook promising molecules on the basis of “poor” SA scores. Overall, we demonstrate that treating retrosynthesis models directly as an oracle is feasible and is an alternative approach to existing methods. To date, optimizing directly for retrosynthesis solvability is minimally used because querying these models can be computationally intensive. As such, generative models with insufficient sample efficiency may need to make a prohibitive number of calls (equating to compute time and cost) to these oracles before successfully optimizing the design objective. On the other hand, sample efficiency has become a core research topic since the release of the Practical Molecular Optimization benchmark^12^ highlighting this problem. Since then, very recent models have pushed sample efficiency with more and more state-of-the-art performances reported on benchmarks.^16,95–99^ As a result, we envision the approach of directly optimizing for retrosynthesis solvability will become more widely adopted.

## Data availability

The data and code is released in the Saturn repository: https://github.com/schwallergroup/saturn.

## Author contributions

J. G. proposed the idea. J. G. and P.S. conceptualized the project. J. G. developed the approach and integrated the retrosynthesis models, performed all experiments, analyzed the data, and wrote the initial draft of the manuscript. P. S. guided the research direction, provided funding and strategic input on methodology, contributed to the manuscript, and supervised the project.

## Conflicts of interest

There are no conflicts to declare.

## Supplementary Material

SC-016-D5SC01476J-s001
