# Supplementary material for: Directly optimizing for synthesizability in generative molecular design using retrosynthesis models
Source: Chem Sci. 2025 Mar 21;16(16):6943–56. doi: 10.1039/d5sc01476j (PMC11927497; doi:10.1039/d5sc01476j)
Supplement: SC-016-D5SC01476J-s001 [file SC-016-D5SC01476J-s001.pdf]

Supplementary Information (SI) for Chemical Science.
This journal is © The Royal Society of Chemistry 2025

Journal Name

ARTICLE TYPE

Cite this: DOI: 00.0000/xxxxxxxxxx

Supporting Information: Directly Optimizing for Synthesizability in Generative Molecular Design using Retrosynthesis Models†

Jeff Guo,*[a,b] and Philippe Schwaller*[a,b]

1 Supporting Information

The Supporting Information contains details on the procedure we took to reproduce RGFN's[1] oracle as the code is not released. In addition, we report the computational resources used, how Saturn was pre-trained, AiZynthFinder and Syntheseus execution details, and example synthesis routes of generated molecules.

2 Compute Resources

All experiments were run on a single workstation with an NVIDIA RTX A6000 GPU 48GB memory and AMD Ryzen 9 5900X 24-Core CPU. 48GB GPU memory is not required. QuickVina2-GPU-2.1[2–4] with 'thread' = 8,000 (following the RGFN[1] work) takes up to 12GB GPU memory. We further note that Saturn's wall times reported in the main text are longer than actually required as we always run 2-4 experiments in parallel, which share the workstation's resources, but makes the *total* wall time less.

3 Saturn Pre-training Details

This section contains the exact protocol used for Saturn pre-training on ChEMBL 33[5] and ZINC 250k[6]. The details and pre-trained models are taken from the original Saturn[7] paper and included here.

3.1 ChEMBL 33

Each step is followed by the SMILES remaining after the filtering step.

1. Download raw ChEMBL 33 - 2,372,674
2. Standardization (charge and isotope handling) based on `https://github.com/MolecularAI/ReinventCommunity/blob/master/notebooks/Data_Preparation.ipynb`. All SMILES that could not be parsed by RDKit were removed - 2,312,459
3. Kept only the unique SMILES - 2,203,884
4. Tokenize all SMILES based on REINVENT's tokenizer: `https://github.com/MolecularAI/reinvent-models/blob/main/reinvent_models/reinvent_core/models/vocabulary.py`
5. Keep SMILES $\leq$ 80 tokens - 2,065,099
6. 150 $\leq$ molecular weight $\leq$ 600 - 2,016,970
7. Number of heavy atoms $\leq$ 40 - 1,975,282
8. Number of rings $\leq$ 8 - 1,974,522
9. Size of largest ring $\leq$ 8 - 1,961,690
10. Longest aliphatic carbon chain $\leq$ 5 - 1,950,213
11. Removed SMILES containing the following tokens (due to undesired chemistry and low token frequency): [S+], [C-], [s+], [O], [S@+], [S@@+], [S-], [o+], [NH+], [n-], [N@], [N@@], [N@+], [N@@+], [S@@], [C+], [S@], [c+], [NH2+], [SH], [NH-], [cH-], [O+], [c-], [CH], [SH+], [CH2-], [OH+], [nH+], [SH2] - **1,942,081**

The final vocabulary contained 37 tokens (2 extra tokens were added, indicating <START> and <END>).

The Mamba model has 5,265,920 parameters. The hyperparameters are the default parameters in the code base.

The pre-training parameters were:

1. Max training steps = 20 (each training step entails a full pass through the dataset)
2. Seed = 0
3. Batch size = 512
4. Learning rate = 0.0001
5. Randomize[8] every batch of SMILES

The following checkpoint was used: Epoch 18, NLL = 32.21, Validity (10k) = 95.60%.

[a] *École Polytechnique Fédérale de Lausanne (EPFL)*
[b] *National Centre of Competence in Research (NCCR) Catalysis*
† Supplementary Information available: [details of any supplementary information available should be included here]. See DOI: 00.0000/00000000.

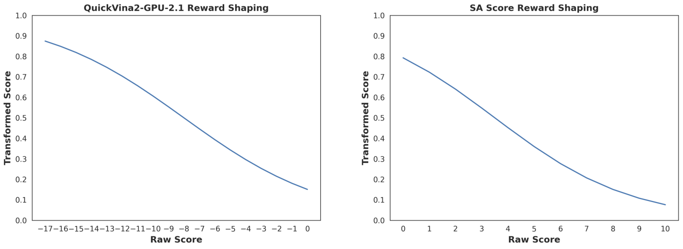

Fig. 1 Saturn reward shaping functions for QuickVina2-GPU-2.1 and SA score.

3.2 ZINC 250k

ZINC 250k[6] was downloaded and used as is.

The pre-training parameters were:

1. Training steps = 50 (each training step entails a full pass through the dataset)
2. Seed = 0
3. Batch size = 512
4. Learning rate = 0.0001
5. Train with SMILES randomization[8] (all SMILES in each batch was randomized)

The final vocabulary contained 66 tokens (2 extra tokens were added, indicating <START> and <END>).

The Mamba model has 5,272,832 parameters (slightly larger than ChEMBL 33 model because the vocabulary size here is larger). The following checkpoint was used: Epoch 50, NLL = 28.10, Validity (10k) = 95.20%.

4 Saturn General Details

4.1 Saturn Reward Shaping

This section contains details on the reward shaping functions used such that the objective functions: $R_{RGFN}, R_{All\ MPO}, R_{Double\ MPO} \in [0,1]$. Fig. 1 shows the functions for QuickVina2-GPU-2.1[2–4] and SA score[9]. QED[10] values were taken as is, and not subjected to reward shaping. AiZynthFinder[11–13] returns 0 for not solved and 1 for solved. Given a molecule, all oracle evaluations are aggregated via a weighted product and a single scalar value is returned as the reward:

$$R(x) = \left[\prod_i p_i(x)^{w_i}\right]^{\frac{1}{\sum_i w_i}} \quad (1)$$

x is a SMILES[14], i is the index of an oracle given many oracles (MPO objective), p_i is an oracle, and w_i is the weight assigned to the oracle (1 for all oracles in this work).

4.2 GraphGA-augmented Experience Replay

Saturn[7] uses experience replay to enhance sample efficiency. GraphGA[15] can be applied on the replay buffer (stores the highest rewarding molecules generated so far) by treating the replay buffer as the parent population. Crossover and mutation operations then generate new molecules. For all the results in this work, activating the GA decreases the AiZynthFinder solve rate relative to no GA. This is because the generated molecules are not being sampled from the model itself (which is *learning* to generate AiZynthFinder solvable molecules). What is gained in return is diversity recovering (as found in the original Saturn[7] work). This can be advantageous since the RGFN[1] work defines **Discovered Modes** as the number of Modes (<-10 docking score) which also have < 0.5 Tanimoto similarity to every other mode. By activating the GA, more Modes are generally found, relative to no GA.

4.3 Saturn Batch Generation

Saturn[7] generates SMILES[14] in batches of, at maximum, 16. We emphasize again that the small batch size is in anticipation of leveraging high-fidelity oracles which may necessitate a small batch size in the event that each molecule needs to be put on a GPU. In a follow-up work, we have shown that in fact, a larger batch size, which makes the sampling behavior more exploratory, can be beneficial when directly optimizing for retrosynthesis models[16]. Internally, there is an oracle caching mechanism such that repeat generated SMILES are not sent for oracle evaluation, and instead, the reward is retrieved from the cache. Saturn's sample efficiency comes from the local exploration of chemical space, such that, at adjacent epochs, identical SMILES can be generated. The effect is that at each generation epoch, sometimes only a few *new* (not generated before) SMILES are generated. In the main text Fig. 4, some batches have 0% solve rate by AiZynthFinder. These are batches that only have a few new SMILES that happen not to be solvable. If one new SMILES is generated, it being unsolvable equates to 0% solve rate.

5 Reproducing RGFN's Oracle

This section contains the steps we took to reproduce RGFN's[1] ATP-dependent Clp protease proteolytic subunit (ClpP) docking case study as faithfully as we could.

Target Preparation. Following Supporting Information C.1 of the RGFN paper, we downloaded the 7UVU ClpP crystal structure here: `https://www.rcsb.org/structure/7UVU`. All molecules (complexed inhibitors, solvents, etc.) were removed, keeping only two monomeric units. Two structures were saved: The apo protein (no other molecules present) and the reference ligand. **The following step differs from RGFN**: the apo protein was processed with PDBFixer[17] to fix missing atoms and residues. We performed this step because errors were thrown during docking when using the raw apo protein structure.

Docking Details. We implement QuickVina2-GPU-2.1[2–4] following the instructions in the GitHub repository here: `https://github.com/DeltaGroupNJUPT/Vina-GPU-2.1`. The reference ligand structure that was saved out in the previous step is used here to define the docking box. Specifically, the average coordinates of the ligand denote the docking centroid. **The following *may* differ from RGFN**: We define the docking box as 20 Å x 20 Å x 20 Å as it was unclear how it should be defined based on RGFN's protocol. This box size has worked on many other protein targets[18] when docking with AutoDock Vina[2] which is the

predecessor of QuickVina2-GPU-2.1.

Docking Workflow. Following RGFN's protocol, QuickVina2-GPU-2.1 used the following parameters: 'thread' = 8,000 with 'search depth' = "heuristic" which is the default. Next, all ligands were docked following RGFN's workflow:

1. Start with batch of generated SMILES from Saturn
2. Canonicalize the SMILES
3. Convert to RDKit Mol objects
4. Protonate the Mols
5. Generate 1 (lowest energy) conformer using 'ETKDG'[19]
6. Minimize energy with the Universal Force Field (UFF)[20]
7. Write out the conformers as 'PDB' files
8. Using Open Babel[21], convert the 'PDB' to 'PDBQT' format
9. Execute QuickVina2-GPU-2.1 docking

Protocol Validation. We make further efforts to ensure the oracle is as faithful as possible to RGFN's implementation. When executing QuickVina2-GPU-2.1, if a seed is not specified, a random seed is used. It is unclear if a seed was set in the RGFN[1] work. In our experiments, the seed is 0. We re-dock the reference ligand and find that the pose is similar to Figure 15 in the RGFN work. However, the docking score we obtain is -9.2 whereas the RGFN work reports -10.31. Subsequently, we execute docking 100 times (letting QuickVina2-GPU 2.1 select the random seed) and observed that seed = 448029751 gives a similar pose to RGFN's pose and yields a docking score of -10.1. We additionally found that seed = 1920393356 yields a docking score of -10.3 but the pose is reflected. Finally seed = 673697018 yields a docking score of -8.2 and is a completely different pose. It is intractable to try every seed.

Therefore, we end this section by stating that it is hard to say if we *exactly* re-implement RGFN's[1] docking oracle. However, we believe it still enables us to convey the primary message of our work: retrosynthesis models can be directly treated as an oracle and be explicitly optimized for during generation.

6 AiZynthFinder

AiZynthFinder[11–13] was used as is, without modification. The source code was cloned from the GitHub repository here: `https://github.com/MolecularAI/aizynthfinder`. The environment and package were installed following the README. Following the documentation here: `https://molecularai.github.io/aizynthfinder/`, we downloaded the public data and used AiZynthFinder as is. We consider a molecule AiZynthFinder "solvable" if the "is_solved" flag is True. This flag denotes whether the top scored (accounting for tree depth and fraction of building blocks in stock)[12,13] is solved.

7 Experiment 2: Directly optimizing for synthesizability using AiZynth Additional Details

7.1 AiZynthFinder Routes

The AiZynthFinder solved routes for the 8 example molecules shown in Fig. 3 in the main text are shown here. The All MPO routes (Fig. 2) are generally shorter than the Double MPO routes (Fig. 3). This suggests that enforcing QED and SA score also implicitly makes the predicted forward syntheses shorter. We note that it is possible to design an objective function that also aims to generate short paths by rewarding short paths. We do not explore this here and leave it for future work.

7.2 Supplementary Results

In this section, supplementary results are reported which aim to address/provide evidence for three points:

1. Effect of increasing the oracle budget when optimizing AiZynthFinder
2. *Jointly* optimizing QED with docking score is *considerably* more difficult than just optimizing docking score
3. Optimizing SA score *can* be a better allocation of computational resources

Increasing the oracle budget leads to notably increased wall times. In the main text results, $R_{All\ MPO}$ does not find that many Modes. We investigate the effect of increasing the oracle budget (Table 1) with and without the GA activated (which recover diversity so as to satisfy the Modes criterion that Modes must have < 0.5 Tanimoto similarity with other Modes). Note that we also ran the experiment with an oracle budget of 1,000 (as in the main text experiments) with the GA activated for completeness. Comparing the results with an increased oracle budget of 5,000, more Modes are found but the wall time is *notably* higher. With 5x the oracle budget (5,000 compared to 1,000 in the main text), one may expect 5x the wall time (12-13 hours) but the wall time is around 18 hours. The reason is due to Saturn's sampling behavior which locally explores chemical space[7]. This leads to batches of repeat molecules which are not scored by the oracle but means it takes more iterations to exhaust the oracle budget. The parameters of Saturn could be changed to loosen this local exploration behavior such as increasing batch size from 16 but we do not explore this. We demonstrate the application of Saturn out-of-the-box and also in anticipation of leveraging high-fidelity oracles which may necessitate a small batch size in the event that each molecule needs to be put on a GPU. As a consequence of this, many repeat molecules are generated, which do not impose an oracle call as the reward is retrieved from an oracle cache, but makes the sampled batch (new molecules) smaller. Consider batches of 1 molecule and 4 molecules. This can take a similar wall time as molecules can be chunked, thus benefiting from multi-threading. The overhead of initializing an oracle can also be non-negligible, such that it is the bottleneck between the wall time to score 1 or 4 molecules. This could be mitigated, for example, by using a faster retrosynthesis model which can come

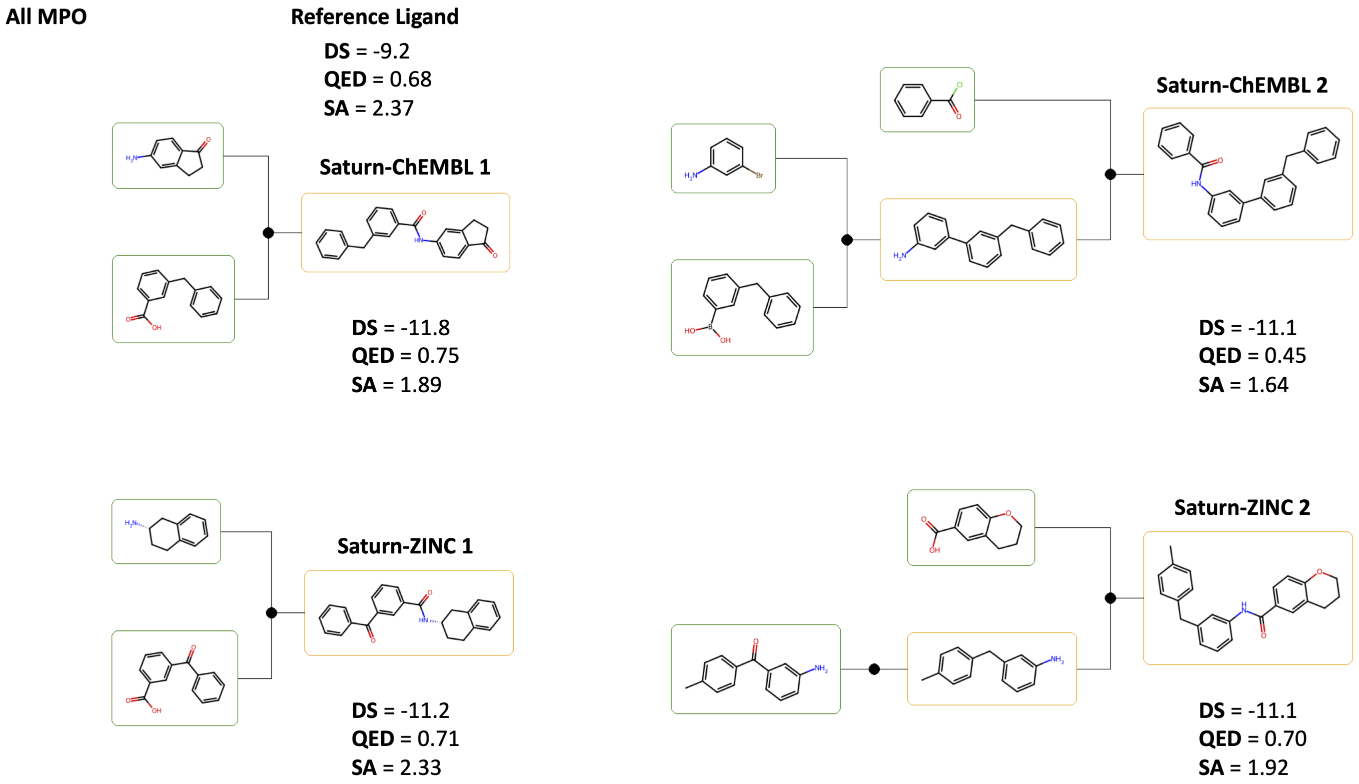

Fig. 2 AiZynthFinder solved routes (top-2–scoring) for All MPO example molecules.

with advantages and disadvantages[22,23] and/or parallelization. Finally, we highlight that *deactivating* the GA will likely lead to higher Yield and AiZynthFinder solve rate, as shown in the main text. We reiterate that activating the GA was to satisfy the Mode metric.

***Jointly* optimizing QED with docking score is *considerably* more difficult than just optimizing docking score.** RGFN[1] reports their mean and standard deviation of QED values as 0.23 ± 0.04. The relatively low QED values suggest that the model is exploiting the docking algorithm as shown in the main text. To show that *jointly* optimizing QED and docking is a *considerably* more difficult task, we first cross-reference the results for $R_{Double\ MPO}$ (Table 1) where the Modes and Yield are notably higher than $R_{All\ MPO}$. Next, we cross-reference the results when QED is *not* being optimized (Table 1 last row). The Yield is *much* higher (molecules with docking score < -10) but the QED values are similar to RGFN, which again, suggests the docking algorithm is being exploited.

Optimizing SA score *can* be a better allocation of computational resources. SA score[9] is correlated with AiZynthFinder solve rate[24]. In the main text Fig. 4, we empirically demonstrate this, as 56 seconds of fine-tuning a pre-trained model that has *never* seen an AiZynthFinder solved molecule, results in a model that generates molecules almost all solvable. The natural next question is, would simply optimizing SA score be a better allocation of computational resources (as is commonly done)? Under the same wall time, many more queries to SA score can be made because it is computationally cheap. Correspondingly, we use the $R_{All\ MPO}$ objective function but omit AiZynthFinder (only docking, QED, and SA score) and run the ChEMBL and ZINC pre-trained models for 10,000 oracle calls (Table 1). Firstly, the wall time is about 1 hour longer than running 1,000 oracle calls of AiZynthFinder. Next, while a smaller *fraction* of the Modes are AiZynthFinder solvable, the raw number is higher than directly optimizing AiZynthFinder. This reinforces that post-hoc retrosynthesis model filtering is valid and is often what is done in practice[25]. Crucially, the actual percentage of AiZynthFinder solve rate may not *actually* matter. What matters is that a user can reasonably expect a generative model to generate molecules satisfying the objective function within the allotted oracle budget and/or wall time. In this specific example, it does not matter that Saturn-ChEMBL "only" has 33% solve rate when optimizing docking, QED, and SA score (Table 1). Running the 131 Modes through AiZynthFinder can take less than 20 minutes. A user would only care that in 3.5 hours, 43/131 Modes were found that have low docking score, high QED, low SA score, and are AiZynthFinder solvable.

Finally, we wish to be prudent with making definitive statements about whether just optimizing SA score is strictly *better* than including a retrosynthesis model in the objective function. In this section alone, we have highlighted that different retrosynthesis models can have a large impact on wall time[22,23], where faster wall times would narrow the gap between SA score's wall time. Moreover, molecules deemed difficult to synthesize by SA score may actually be straightforward to synthesize. Retrosynthesis models have much more flexibility as the building block stock

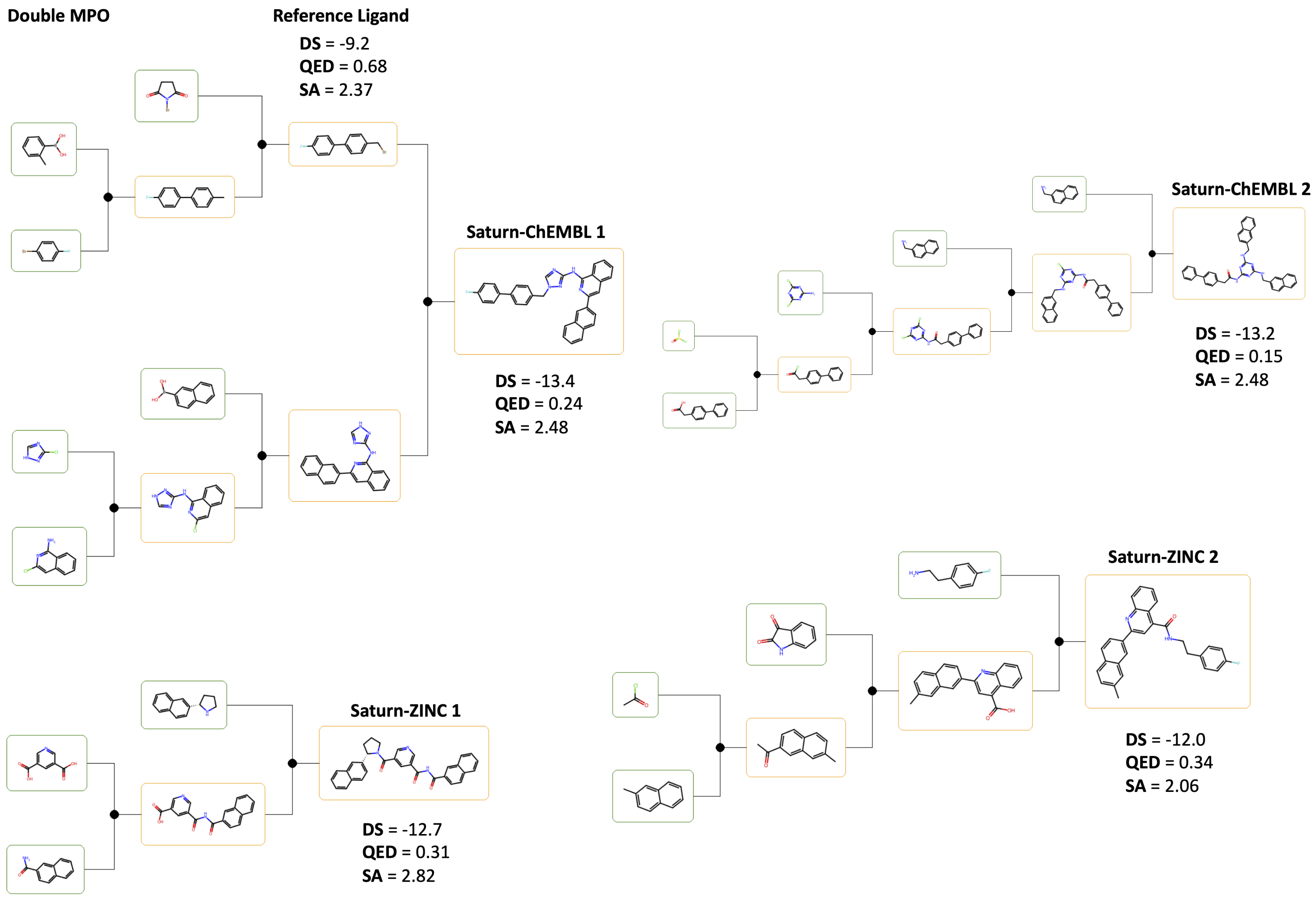

Fig. 3 AiZynthFinder solved routes (top-2-scoring) for Double MPO example molecules.

Table 1 Synthesizability metrics across various Saturn experiments. Metrics are reported for however many Modes are found. For these supplemental results, only the experiments with an oracle budget of 1,000 were run across 10 seeds (0-9 inclusive). Other experiments were only run for one replicate (seed = 0). For the experiments run across seeds, the number after the configuration denotes the number of successful replicates out of 10 (Modes $\geq$ 1). Note that the means and standard deviations are only reported for the experiments with an oracle budget of 1,000 as these were performed in replicates.

Method	Modes (Yield)	Mol. weight (↓)	QED (↑)	SA score (↓)	AiZynth (↑)	Oracle calls (Wall time)
$R_{All\ MPO}$	4 objectives (Docking, QED, SA, AiZynth)					
Saturn-GA-ChEMBL (9)	6 ± 3 (7 ± 4)	390.4 ± 22.9	0.66 ± 0.10	2.23 ± 0.19	0.81 ± 0.21	1,000 (2h 37m ± 12m)
Saturn-GA-ZINC (10)	7 ± 4 (11 ± 8)	368.7 ± 19.3	0.71 ± 0.11	2.01 ± 0.15	0.94 ± 0.08	1,000 (2h 17m ± 8m)
Saturn-ChEMBL	31 (52)	379.6	0.75	2.18	0.81	5,000 (18h 37m)
Saturn-ZINC	88 (252)	366.2	0.85	2.33	0.89	5,000 (14h 58m)
$R_{Double\ MPO}$	2 objectives (Docking, AiZynth)					
Saturn-GA-ChEMBL (10)	53 ± 10 (119 ± 40)	422.3 ± 16.8	0.41 ± 0.05	2.30 ± 0.09	0.78 ± 0.06	1,000 (2h 51m ± 13m)
Saturn-GA-ZINC (10)	26 ± 9 (62 ± 33)	413.8 ± 21.3	0.48 ± 0.06	2.28 ± 0.15	0.86 ± 0.07	1,000 (2h 30m ± 10m)
Saturn-ChEMBL	320 (3131)	446.1	0.36	2.37	0.86	5,000 (17h 41m)
Saturn-GA-ChEMBL	350 (2783)	441.0	0.37	2.62	0.68	5,000 (16h 29m)
Saturn-ZINC	228 (3722)	432.9	0.30	2.45	0.71	5,000 (18h 51m)
Saturn-GA-ZINC	351 (2138)	452.0	0.48	2.69	0.67	5,000 (18h 10m)
$R_{All\ MPO}$ (but without AiZynth)	3 objectives (Docking, QED, SA)					
Saturn-ChEMBL	131 (1738)	350.6	0.88	2.49	0.33	10,000 (3h 37m)
Saturn-ZINC	204 (980)	364.6	0.83	2.64	0.17	10,000 (3h 38m)
R_{RGFN} - Results also shown in the main text	1 objective (Docking)					
Saturn-ChEMBL	369 (8544)	551.5	0.27	2.82	0.28	10,000 (2h 57m)

and reactions can be changed, whereas SA score was designed based on the fixed PubChem corpus[9]. One could even constrain the retrosynthesis model to only include building blocks and reactions that are available in-house, similar to what was done in a collaborative work involving Pfizer[26]. Finally, in the main text, we have shown that *the specific* drug discovery case study performed in this work benefits more from optimizing SA score in place of retrosynthesis models but this should not be taken as a general result. We conversely showed that in a semiconductor case study[27], SA score led to very few synthesizable molecules compared to a retrosynthesis model. Therefore, the only conclusion we make is that optimizing retrosynthesis models explicitly can mitigate out-of-distribution challenges associated with synthesizability heuristics scores.

8 Experiment 3: Directly optimizing for synthesizability using AiZynth starting from an unsuitable training distribution Additional Details

8.1 AiZynthFinder purged ZINC 250k Pre-training Details.

In Experiment 3, we pre-train Saturn on a sub-set of ZINC 250k[6] that *is not* AiZynthFinder[11–13] solvable. The goal is to show that Saturn can *still* optimize for generating molecules that are AiZynthFinder solvable despite being trained on no molecules that can be.

Purged Dataset. We first run AiZynthFinder on the entirety of ZINC 250k on a single workstation with an NVIDIA RTX A6000 GPU 48GB memory and AMD Ryzen 9 5900X 24-Core CPU. The process was run using multi-threading across 12 workers and took 62 hours. We save the unique SMILES (98,110) of all the molecules that *are not* AiZynthFinder solvable. This is the dataset used for pre-training.

Pre-training. Following the same pre-training parameters used in the original Saturn[7] work:

1. Training steps = 100 (each training step entails a full pass through the dataset)
2. Seed = 0
3. Batch size = 512
4. Learning rate = 0.0001
5. Train with SMILES randomization[8] (all SMILES in each batch was randomized)

The final vocabulary contained 57 tokens (2 extra tokens were added, indicating <START> and <END>). This is less than the normal ZINC 250k model (66 tokens) because some tokens are not present in the purged dataset.

The Mamba model has 5,271,040 parameters (less than the normal 250k model because the vocabulary size is smaller). The following checkpoint was used: Epoch 100, NLL = 27.78, Validity (10k) = 92.27% and the training time was 4.7 hours.

9 Experiment 4: Directly optimizing for synthesizability using any retrosynthesis model Additional Details

9.1 Syntheseus

In Experiment 4 in the main text, we extended the retrosynthesis model from AiZynthFinder to RetroKNN[28], Graph2Edits[29], and

RootAligned[30]. We ran these models through the Syntheseus[23] package to show that *any* retrosynthesis model can be directly optimized for. The main difference, other than the model, when compared to AiZynthFinder, is the commercial building blocks set used. For AiZynthFinder, we used the default ZINC[6] building blocks provided in the AiZynthFinder installation. For Syntheseus, we used the 'Fragment' and 'Reactive' sub-sets of ZINC[6] (17,721,980 building blocks).

10 Experiment 5: Generating out-of-distribution of synthesizability heuristics Additional Details

10.1 Wall times

In this section, we report the wall times for all configurations used in the drug discovery and semiconductor case study. The experiment in this section contrasts optimizing for SA score compared to directly incorporating a retrosynthesis model directly in the optimization loop. In addition to reporting the raw compute time values, we also want to convey that we increased the SA score experiments' oracle budgets so that the total wall time of the SA score (with 3,000 oracle calls budget) experiments were comparable to 1,000 oracle calls involving the retrosynthesis model for fairness. Moreover, we note that in the semiconductors case study, we used xTB[31] to compute electronic properties. Technically, after a single-point calculation, both the HOMO-LUMO gap and dipole values we use are obtained but due to an existing sub-optimal implementation, we re-ran xTB to compute the other property. This effectively doubles the xTB time but affects all configurations so the wall time comparisons are valid. In the future, this will be replaced by another module that better handles information flow and extraction. Tables 2 and 3 show the wall times for the drug discovery and semiconductor case studies, respectively. We increased the oracle budget accordingly for the SA score configurations to match the retrosynthesis model configurations. Finally, ZINC runs took longer due to the much larger building block stock (17.7 million).

Drug Discovery Case Study

Configuration	Wall time (seconds)
SA (1000)	590 ± 71
SA (3000)	3473 ± 348
Direct Enamine EU (1000)	3664 ± 770
Direct Enamine US (1000)	3171 ± 268
Direct Sigma-Aldrich (1000)	4938 ± 931
Direct ZINC (1000)	19690 ± 3942

Table 2 Average wall times across 10 seeds for Drug Discovery Case Study. The oracle budget is in parentheses.

Semiconductor Case Study

10.2 Semiconductor Case Study SA Score Distribution

Fig. 4 shows the SA score distribution of various experiment configurations. There is minimal difference between the SA scores of synthesizable and non-synthesizable molecules.

Configuration	Wall time (seconds)
SA (1000)	2684 ± 568
SA (2000)	9557 ± 3958
Direct Enamine EU (1000)	8556 ± 1693
Direct Enamine US (1000)	8310 ± 1185
Direct Sigma-Aldrich (1000)	9960 ± 3315
Direct ZINC (1000)	22792 ± 4225

Table 3 Average wall times across 10 seeds for Semiconductor Case Study. The oracle budget is in parentheses.

10.3 Point-Biserial Correlation

We use point-biserial correlation to measures the correlation between SA scores and synthesizability assessed across all stocks. Synthesizability is given as a binary variable: 0 or 1. A more negative correlation indicates that SA score decreases when synthesizability is 1, i.e., synthesizable. Table 4 shows the correlations. In the drug discovery case study, lower SA scores tend to equate to more synthesizable molecules while in the semiconductor case study, the variables are almost completely uncorrelated.

Stock	Drug Discovery (SA 3000)	Semiconductor (SA 2000)
Enamine EU	-0.378 ± 0.068	-0.072 ± 0.100
Enamine US	-0.397 ± 0.077	-0.040 ± 0.116
Sigma Aldrich	-0.455 ± 0.053	-0.145 ± 0.088
ZINC	-0.512 ± 0.080	-0.013 ± 0.146

Table 4 Average point-biserial correlation across 10 seeds for Drug Discovery (SA 3000) and Semiconductor (SA 2000) Case Studies. The oracle budget is in parentheses.

Notes and references

1 M. Koziarski, A. Rekesh, D. Shevchuk, A. van der Sloot, P. Gaiński, Y. Bengio, C.-H. Liu, M. Tyers and R. A. Batey, *arXiv preprint arXiv:2406.08506*, 2024.

2 O. Trott and A. J. Olson, *Journal of computational chemistry*, 2010, **31**, 455–461.

3 A. Alhossary, S. D. Handoko, Y. Mu and C.-K. Kwoh, *Bioinformatics*, 2015, **31**, 2214–2216.

4 S. Tang, J. Ding, X. Zhu, Z. Wang, H. Zhao and J. Wu, *bioRxiv*, 2023, 2023–11.

5 A. Gaulton, L. J. Bellis, A. P. Bento, J. Chambers, M. Davies, A. Hersey, Y. Light, S. McGlinchey, D. Michalovich, B. Al-Lazikani *et al.*, *Nucleic acids research*, 2012, **40**, D1100–D1107.

6 T. Sterling and J. J. Irwin, *Journal of chemical information and modeling*, 2015, **55**, 2324–2337.

7 J. Guo and P. Schwaller, *arXiv preprint arXiv:2405.17066*, 2024.

8 E. J. Bjerrum, *arXiv preprint arXiv:1703.07076*, 2017.

9 P. Ertl and A. Schuffenhauer, *Journal of cheminformatics*, 2009, **1**, 1–11.

10 G. R. Bickerton, G. V. Paolini, J. Besnard, S. Muresan and A. L. Hopkins, *Nature chemistry*, 2012, **4**, 90–98.

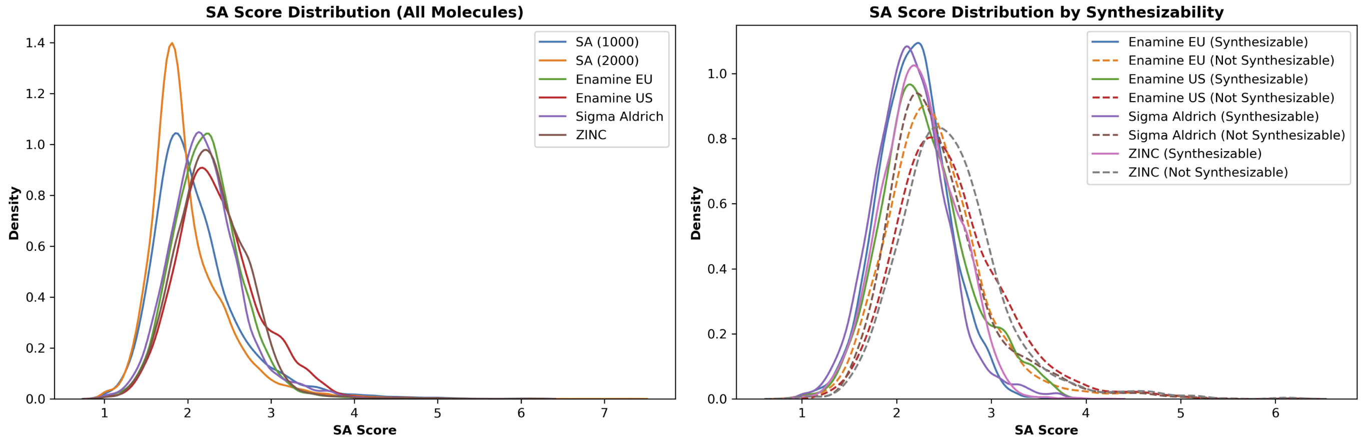

Fig. 4 Semiconductor case study: SA scores distribution. The experimental configurations that optimize for low SA score do successfully generate molecules with low SA score. There is also minimal difference between the SA scores of synthesizable and non-synthesizable molecules.

11 A. Thakkar, T. Kogej, J.-L. Reymond, O. Engkvist and E. J. Bjerrum, *Chemical science*, 2020, **11**, 154–168.

12 S. Genheden, A. Thakkar, V. Chadimová, J.-L. Reymond, O. Engkvist and E. Bjerrum, *Journal of cheminformatics*, 2020, **12**, 70.

13 L. Saigiridharan, A. K. Hassen, H. Lai, P. Torren-Peraire, O. Engkvist and S. Genheden, *Journal of Cheminformatics*, 2024, **16**, 57.

14 D. Weininger, *Journal of chemical information and computer sciences*, 1988, **28**, 31–36.

15 J. H. Jensen, *Chemical science*, 2019, **10**, 3567–3572.

16 J. Guo and P. Schwaller, *arXiv preprint arXiv:2410.11527*, 2024.

17 P. Eastman, M. S. Friedrichs, J. D. Chodera, R. J. Radmer, C. M. Bruns, J. P. Ku, K. A. Beauchamp, T. J. Lane, L.-P. Wang, D. Shukla *et al.*, *Journal of chemical theory and computation*, 2013, **9**, 461–469.

18 J. Guo, J. P. Janet, M. R. Bauer, E. Nittinger, K. A. Giblin, K. Papadopoulos, A. Voronov, A. Patronov, O. Engkvist and C. Margreitter, *Journal of cheminformatics*, 2021, **13**, 1–21.

19 S. Riniker and G. A. Landrum, *Journal of chemical information and modeling*, 2015, **55**, 2562–2574.

20 A. K. Rappé, C. J. Casewit, K. Colwell, W. A. Goddard III and W. M. Skiff, *Journal of the American chemical society*, 1992, **114**, 10024–10035.

21 N. M. O'Boyle, M. Banck, C. A. James, C. Morley, T. Vandermeersch and G. R. Hutchison, *Journal of cheminformatics*, 2011, **3**, 1–14.

22 B. Chen, C. Li, H. Dai and L. Song, International conference on machine learning, 2020, pp. 1608–1616.

23 K. Maziarz, A. Tripp, G. Liu, M. Stanley, S. Xie, P. Gaiński, P. Seidl and M. Segler, *arXiv preprint arXiv:2310.19796*, 2023.

24 G. Skoraczyński, M. Kitlas, B. Miasojedow and A. Gambin, *Journal of Cheminformatics*, 2023, **15**, 6.

25 J. D. Shields, R. Howells, G. Lamont, Y. Leilei, A. Madin, C. E. Reimann, H. Rezaei, T. Reuillon, B. Smith, C. Thomson *et al.*, *RSC Medicinal Chemistry*, 2024, **15**, 1085–1095.

26 A. K. Hassen, M. Sicho, Y. J. van Aalst, M. C. Huizenga, D. N. Reynolds, S. Luukkonen, A. Bernatavicius, D.-A. Clevert, A. P. Janssen, G. J. van Westen *et al.*, *ChemRxiv*, 2024.

27 Q. Yuan, A. Santana-Bonilla, M. A. Zwijnenburg and K. E. Jelfs, *Nanoscale*, 2020, **12**, 6744–6758.

28 S. Xie, R. Yan, J. Guo, Y. Xia, L. Wu and T. Qin, Proceedings of the AAAI Conference on Artificial Intelligence, 2023, pp. 5330–5338.

29 W. Zhong, Z. Yang and C. Y.-C. Chen, *Nature Communications*, 2023, **14**, 3009.

30 Z. Zhong, J. Song, Z. Feng, T. Liu, L. Jia, S. Yao, M. Wu, T. Hou and M. Song, *Chemical Science*, 2022, **13**, 9023–9034.

31 C. Bannwarth, S. Ehlert and S. Grimme, *Journal of chemical theory and computation*, 2019, **15**, 1652–1671.
